# Comparison of the Electrodialysis Performance in Tartrate Stabilization of a Red Wine Using Aliphatic and Aromatic Commercial and Modified Ion-Exchange Membranes

**DOI:** 10.3390/membranes13010084

**Published:** 2023-01-09

**Authors:** Evgeniia Pasechnaya, Kseniia Tsygurina, Maria Ponomar, Daria Chuprynina, Victor Nikonenko, Natalia Pismenskaya

**Affiliations:** 1 Membrane Institute, Kuban State University, 350040 Krasnodar, Russia; 2 Department of Analytical Chemistry, Kuban State University, 350040 Krasnodar, Russia

**Keywords:** electrodialysis, tartrate stabilization, aliphatic, aromatic, ion-exchange membrane, modification, layer by layer, fouling

## Abstract

The application of electrodialysis for tartrate stabilization and reagent-free acidity correction of wine and juices is attracting increasing interest. New aliphatic membranes CJMC-3 and CJMA-3 and aromatic membranes CSE and ASE were tested to determine their suitability for use in these electrodialysis processes and to evaluate the fouling of these membranes by wine components for a short (6–8 h) operating time. Using IR spectroscopy, optical indication and measurement of surface contact angles, the chemical composition of the studied membranes, as well as some details about their fouling by wine components, was clarified. The current–voltage charsacteristics, conductivity and water-splitting capacity of the membranes before and after electrodialysis were analyzed. We found that in the case of cation-exchange membranes, complexes of anthocyanins with metal ions penetrate into the bulk (CJMC-3) or are localized on the surface (CSE), depending on the degree of crosslinking of the polymer matrix. Adsorption of wine components by the surface of anion-exchange membranes CJMA-3 and ASE causes an increase in water splitting. Despite fouling under identical conditions of electrodialysis, membrane pair CJMC-3 and CJMA-3 provided 18 ± 1 tartrate recovery with 31 · 10^−3^ energy consumption, whereas CSE and ASE provided 20 ± 1% tartrate recovery with an energy consumption of 28 · 10^−3^ Wh, in addition to reducing the conductivity of wine by 20 ± 1%. The casting of aliphatic polyelectrolyte films on the surface of aromatic membranes reduces fouling with a relatively small increase in energy consumption and approximately the same degree of tartrate recovery compared to pristine CSE and ASE.

## 1. Introduction

Quality grape wine is an integral part of the food preferences of most of the world’s population. The industrial production of this drink comprises more than 13,500 million decalitres per year [1]. Moreover, centuries-old traditional technologies are constantly being improved. Thus, the successful application of membrane technologies [2,3,4] has led to a sharp increase in the volume of exports and imports of wine [5,6]. The use of membrane technologies, firstly, makes it possible to reduce production costs due to the organization of a continuous and controlled automatic production mode, in addition to reducing energy costs, process duration and wine losses. Secondly, membrane technologies contribute to a reduction in secondary emissions into the environment, for example, due to the abandonment of reagent methods for stabilizing wine [2]. Stability, in this case, means the absence of any undesirable physical, chemical or organoleptic changes during a certain period of wine storage [7].

One of the causes of clouding and precipitation in wines is the formation of poorly soluble potassium and calcium tartrates [8]. Traditional methods of tartrate stabilization include abrupt or prolonged cooling of wine [9,10,11], inhibition of the process of crystallization of tartrate salts by introducing metatartaric acid [12] or potassium polyaspartate [13] and coagulation of tartrate-containing (and other) complexes or colloidal particles with their subsequent sedimentation or filtration [13]. In this case, mannoproteins [11,14], bentonite [13], carboxymethyl cellulose [12,15] and other organic substances [11] are introduced into the wine materials. The use of ion-exchange resins is the cheapest method of tartrate stabilization [16]. However, this method sometimes causes a change in the ionic composition of wine because resins adsorb valuable components, in addition to tartrates or metal ions [17]. Furthermore, the pretreatment and regeneration of resins requires the use of chemicals [18,19]. On the contrary, electrodialysis (ED) stabilization does not involve the addition of foreign substances and does not lead to a loss of wine quality compared to cold treatment [20] or other reagent methods [21,22]. That is why ED has been approved for commercial use as an alternative technology for tartrate wine stabilization [23,24,25,26].

Systematic research on the use of ED in winemaking began in the 1970s [27,28,29] and led to the development of automated technology [30] using conventional ED [31] or conventional ED combined with bipolar membrane electrodialysis [32,33]. Under the influence of an electric field, anions (chlorides, hydrotartrates, HT^−^, tartrates, T^2−^, etc.) are transferred through anion-exchange membranes (AEM), and cations (mainly K^+^) are transferred through cation-exchange membranes (CEM). The desalination degree of wine in the desalting circuit is estimated by electrical conductivity, the value of which depends on the type of wine. desalination degree is adjusted empirically for each type of wine [34]. Thus, the electrical conductivity should be reduced by 15–20% for young wines, 5–15% for old wines [35] and 20–30% for dessert wines [35,36] in order to achieve the optimal concentration of tartaric anions. A greater change in the chemical composition, in particular the removal of tartaric acid anions, degrades the quality of the wine [27,37,38]. The process of ED stabilization of wine should not lead to a change in the alcohol concentration of more than 0.1% vol., a decrease in the pH value by more than 0.25 units and a change in volatile acidity (in terms of H_2_SO_4_) of more than 0.09 g/L compared to the initial values, or to the loss of polysaccharides and polyphenols (catechins, leucoanthocyanins and anthocyanins), which exhibit antioxidant and antibacterial activity [39,40]. It has been shown [41] that ED tartrate stabilization significantly reduces the loss of polyphenols compared to traditional methods but does not completely eliminate them.

It is known [42,43] that polyphenols, as well as complexes and colloidal particles formed by polyphenols and other components of wine, actively interact with ion-exchange membranes. These are electrostatic interactions of negatively charged fixed groups of cation-exchange materials with the flavylium cation of anthocyanins that acquire a positive charge at pH 2.8–3.5 characteristic of wine [42,43]. Positively charged fixed groups of anion-exchange materials interact with negatively charged complexes or colloidal particles, which are formed by anthocyanins, proanthocyanidins, proteins and anions of tartaric [44] and other organic acids. In addition, π-π (stacking) interactions occur between the aromatic rings of polyphenols and compounds that are part of ion-exchange membranes [45]. Numerous hydrogen bonds take place between oxygen and hydrogen. These elements are found both in the organic components of wine (polyphenols, organic polybasic acids, amino acids, proteins, saccharides, etc.) and in the fixed groups and organic materials contained in AEM and CEM. The accumulation of wine components (primarily anthocyanins and proanthocyanidins) takes place in the volume and on the surface of ion-exchange membranes [42,46]. In addition to the loss of valuable components of wine, fouling leads to a decrease in the ion-exchange capacity, conductivity, selectivity and mechanical stability of membranes [47]. Similar problems arise with the use of ED for the reagentless deacidification of wine and fruit juices [43] or the use of ED during the final stage of wastewater treatment in wineries [45,46]. The market potential of these hybrid technologies is promising for the production of crude extracts with a high phenolic or calcium tartrate content [48,49].

AEM and CEM are most often used in the food industry with an aromatic matrix [43,50], which enters into the all mentioned above types of interactions with wine components. Furthermore, an increasing number of relatively inexpensive commercial ion-exchange membranes with an aliphatic matrix have recently been introduced. These include, for example, membranes manufactured by Fujifilm (Tilburg, The Netherlands) and Chemjoy Polymer Materials Co., Ltd. (Hefei, China). In addition, well-known manufacturers of aromatic membranes, such as Astom (Yamaguchi, Japan), are upgrading widely used membranes [51], for example, CMX and AMX. The listed membranes are increasingly used for various purposes [52,53,54,55,56]. However, their behavior in ED wine stabilization is still unknown. In addition, we cannot predict the fouling of new membranes by wine components in advance, because knowledge about the chemical composition and structure of CJMC-3, CJMA-3, CSE and ASE is fragmentary.

The purpose of this study was to compare the behavior of new aliphatic and aromatic ion-exchange membranes during tartrate stabilization of wine using electrodialysis, as well as to evaluate their fouling with wine components. We used this knowledge to counter fouling by modifying the surface of the membranes. In particular, giving the AEM and SEM surface an electric charge opposite to that of the foulant [57,58] and changing its hydrophilic/hydrophobic balance [57,58,59] was tested. These methods have proven effective in counteracting fouling from bacteria, proteins and some aromatic acids. However, as far as we know, they have not yet been used to extend the life cycle of ion-exchange membranes in the electrodialysis processing of wine and juices.

## 2. Materials and Methods

### 2.1. Membranes

Hefei Chemjoy Polymer Materials Co., Ltd., Hefei, China, manufactures CJMA-3 and CJMC-3 homogeneous membranes. The basis of their ion-exchange matrix is polyvinylidene fluoride [60], which is crosslinked using substances containing aromatic rings [61]. Detailed information about their conductivity, diffusion permeability and selectivity is provided in [62,63]. Homogeneous membranes ASE and CSE are manufactured by Astom (Yamaguchi, Japan) [51]. The manufacturer does not provide information on the chemical structure of these membranes.

CJMC-3 and CSE cation-exchange membranes contain sulfonate fixed groups. The fixed groups of CJMA-3 and ASE anion-exchange membranes are mainly quaternary amines [51,63]. IR spectra of these membranes are presented in Supplementary Materials (SM) Figures S1–S4 and are discussed in Section 3.2.2.

Table 1 lists some characteristics of the studied membranes.

Heterogeneous membranes MK-40 and MA-41 (Shchekinoazot, Shchekino, Russia) were used as auxiliary membranes. Their characteristics are detailed in [62,63].

### 2.2. Solutions

“Tristoriya” dry red wine (Tristoriya Appellation, Novorossiysk, Krasnodar Territory, Russia) was the base a model solution. This wine was made in 2021 from Cabernet Sauvignon (50%) and Syrah (50%) grapes. The composition of the wine is listed in Table 2. Some of the components were obtained using chromatographic methods (Krystall–2000M, Chromatech-Analytic, Yoshkar-Ola, Russia), and non-volatile compounds were determined by the capillary electrophoresis method (Kapel–105M, NPF Lumex, Saint Petersburg, Russia). Note that wine contains ions Cl^−^ (193 ± 5 mg/L), SO_4_^2−^ (969 ± 5 mg/L) and K^+^ (1281 ± 5 mg/L), as well as molecules and anions of tartaric acid (1281 ± 5 mg/L) and other organic acids. Sugar and ethyl alcohol are 10 ± 1%wt and 11 ± 1%vol, respectively.

The concentrations of anthocyanin (Ant) and proanthocyanidin (PAC) polymers in the wine were equal to 77 ± 3 mg/L (in terms of cyanidin-3-glucoside) and 235 ± 3 mg/L, respectively. They were determined using an ECOVIEW UV-1800 spectrophotometer (Shanghai Mapada Instruments Co., Ltd., Shanghai, China) following the procedures described in [64]. This wine had already been subjected to tartrate stabilization. That is why 2000 mg/L of tartaric acid and 400 mg/L of potassium chloride were added to imitate the composition of the wine material before tartrate stabilization. The total content of tartrates, chlorides and potassium cations in this case corresponded to the average content of these substances in wine materials. The conductivity and pH of the model solution were 2.67 ± 0.1 mS/cm and 3.30 ± 0.05, respectively. This indicator is also in a typical range for wine materials before tartrate stabilization [31].

Auxiliary solutions for electrodialysis and solutions used to study the characteristics of membranes were prepared using sodium chloride (NaCl) and tartaric acid (H_2_T) crystals of analytical grade (JSC Vekton, Saint Petersburg, Russia), as well as chemical-purity-grade crystals of potassium chloride (Mikhailovsky Plant of Chemical Reagents, Barnaul, Russia). All solutions were prepared using distilled water with an electrical resistance of 300 ± 10 kΩ cm and pH of 5.04 ± 0.01 at 25 °C.

### 2.3. Electrodialysis Removal of Tartrates

**Figure 1:**
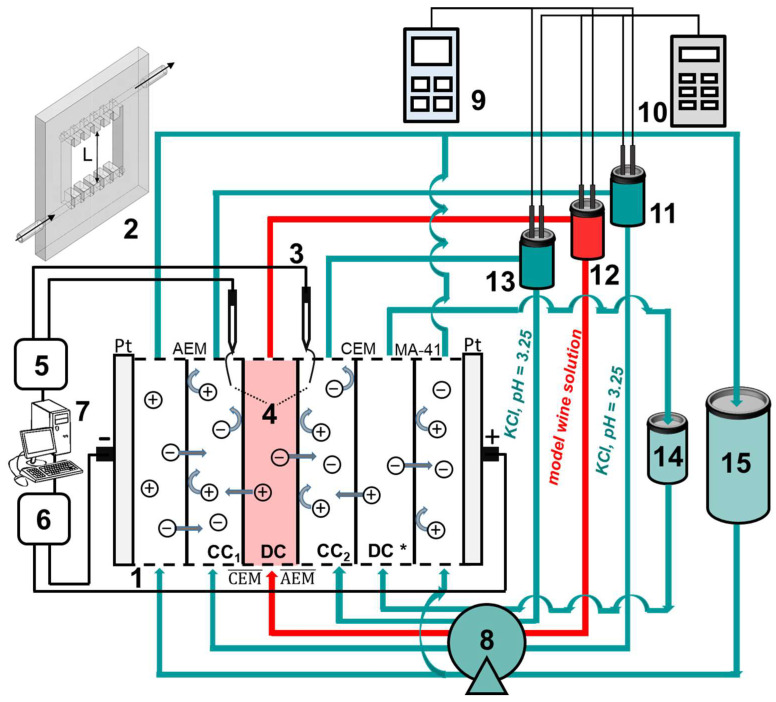
Scheme of the experimental setup: lab-scale ED cell (1) with membranes under study ($\bar{\mathrm{AEM}}$ and $\bar{\mathrm{CEM}}$) and auxiliary membranes (CEM is identical to $\bar{\mathrm{CEM}}$, and AEM is identical to $\bar{\mathrm{AEM}}$); a plastic frame with comb inlet and outlet devices that separate the membranes (2); measuring Ag/AgCl electrodes (3) placed in microreservoirs with Luggin capillaries (4); AKIP V7-78-1 digital multimeter (Siglent, Shenzhen, China) (5); Keithley 2200-60-2 programmable power supply (Keithley Instruments, Cleveland, USA) (6); personal computer (7); Heidolph Pumpdrive 5001 peristaltic pump (Heidolph Instruments GmbH & Co., Schwabach, Germany) (8); Expert-001 pH meter (Econix-Expert Ltd., Rumyantsevo, Russia) (9); Expert-002 conductometer (Econix-Expert Ltd., Rumyantsevo, Russia) (10); intermediate tanks in the desalination (DC), concentration (CC) and electrode (EC) compartment circuits (11–15, respectively).

Tartrate stabilization of the model solution was carried out using a six-compartment laboratory ED cell (Figure 1).

The $\bar{\mathrm{AEM}}$ and $\bar{\mathrm{CEM}}$ membranes under study formed a desalination compartment (DC) through which the model solution was pumped. Auxiliary membranes AEM and CEM were used to limit the adjacent concentration compartments (CC_1_ and CC_2_). These membranes are identical to those under study. Auxiliary membrane MA-41 separated one more desalination compartment (DC *) from the anode compartment. Each membrane had a polarizable area equal to 7.29 ± 0.01 cm^2^. Solution desalination length (*L*) was equal to 2.70 ± 0.01 cm, and the intermembrane distance (*h*) was 0.66 ± 0.01 cm. Before the start of the experiment, all circuits, except the DC circuit, were filled with KCl solution (0.4 g/L). The volumes of circulating solutions were equal to: 150 ± 1 mL (DC, CC_1_ and CC_2_), 1000 ± 1 mL (DC *) and 5000 ± 1 mL (the electrode compartments, EC). The average velocity of the pumped solution flow (*V*_0_) was equal to 0.42 cm/s (DC, DC *, CC_1_ and CC_2_) and 1.68 cm/s (EC). Electrodialysis was carried out at a current density of 1.22 mA/cm^2^. The potential drop was measured between the Luggin capillaries. The capillaries were mounted in microreservoirs with Ag/AgCl electrodes (3). The change in the conductivity and pH of solutions in the circuits of DC, CC_1_ and CC_2_ during ED was measured using conductometric sensors and combined pH electrodes immersed in tanks (11,12,13). The experiment was terminated when the conductivity of the model solution decreased by 25 ± 1% compared to the initial value. The concentrations of K^+^ cations and Cl^−^ anions and the total content of tartrates in the DC, CC_1_ and CC_2_ circuits before and after electrodialysis were determined using a DIONEX ICS-3000 (USA) chromatographic system with a conductometric detector and a background signal suppression unit.

The degree of demineralization and concentration of solutions (*γ*) in the DC, CC_1_ and CC_2_ circuits, as well as the degree of removal of tartrates (*γ_T_*) from the DC circuit, was calculated (in %) according Formulas (1) and (2):

(1) $$\gamma =\frac{\kappa_{t}-\kappa_{0}}{\kappa_{0}}\;\cdot 100\%$$

(2) $$\gamma_{T}=\frac{\left(c_{0}^{T}-c_{t}^{T}\right)}{c_{0}^{T}}\cdot 100\%$$

Indices 0 and *t* correspond to the values of the conductivity (*κ*) of the solution and the total molar concentration of tartrates (*c^T^*) before and during ED for time *t*. A positive *γ* value indicates an increment of ions concentration, whereas a negative value of *γ* is an indicator of decreasing of ion concentration. Fluxes of ions through the membranes were calculated according to Formula (3):

(3) $$j_{i}=\frac{n}{S\cdot t}$$

where *n* is the quantity of matter (in mol), *S* is a membrane surface (in m^2^) and *t* is ED duration (in s). Index *i* corresponds to the type of transported ion.

Energy consumption (in W · s or Joule) was calculated according to Formula (4):

(4) W=I·Δφ·t

where *I* is the current (in A), Δ*φ* is the potential drop (in V) and *t* is ED duration (in s). The number of transported electric charges was determined according to Formula (5):

(5) Q=I·t

### 2.4. Membrane Fouling Study

Before and after electrodialysis, current–voltage curves of the membranes under study were obtained using an Autolab PGSTAT-100 electrochemical complex (Metrohm Autolab B.V., Kanaalweg, The Netherlands). The current sweep rate was 0.02 mA/cm^2^. Before the experiment, the Luggin capillaries were brought to the studied membrane (e.g., CSE), and the adjacent membrane in the DC (e.g., ASE) was replaced with an auxiliary MA-41 membrane. In the case of the study of anion-exchange membranes, the cation-exchange membrane in DC was replaced with an auxiliary MK-40 membrane. The 0.02 M NaCl solution was pumped through all circuits of the electrodialysis cell.

The theoretical limiting current was calculated using the Lévêque equation [65] according to Formula (6):

(6) $$i_{\lim}^{Lev}=\frac{z_{1}FDc_{1}^{0}}{h(T_{1}-t_{1})}\left[1.47{\left(\frac{h^{2}V_{0}}{LD}\right)}^{1/3}\right].\;$$

where *z*_1_ and $c_{1}^{0}$ are the charge number and the molar concentration of the counterion, respectively, at the inlet of the DC compartment; *T*_1_ and *t*_1_ are the effective transport number of the counterion in the membrane (assumed to equal 1 in calculations of *i_lim_^Lev^*) and the counterion transport number in the solution, respectively; *F* is the Faraday constant; and *D* is the binary electrolyte diffusion coefficient.

The conductivity of membranes before and after electrodialysis was determined in 0.1 M NaCl solution by a differential method using a clip cell [66,67]. The measurements were taken at an alternating current frequency of 1 kHz using an AKIP 6104 immittance meter (B + K Precision Taiwan, Inc., New Taipei City, Taiwan).

Optical images of membranes before and after electrodialysis were obtained using a SOPTOP CX40M microscope (Yuyao, Zhejiang, China).

FTIR spectra of the membranes were obtained using a Vertex-70 spectrometer (Bruker Optics, Ettlingen, Germany) and OPUS™ software (Cooperative Library Network, Berlin–Brandenburg, Germany).

The sessile drop method was used to determine the contact angle between a wet membrane surface and a water drop lying on its surface. Details of the experiments are described in [68].

### 2.5. Membrane Surface Modification

A thin film of LF-4SK (NPO Plastpolimer, Saint Petersburg, Russia) coated the surface of an anion-exchange membrane. Aliphatic sulfonated fluoropolymer LF-4SK is an analogue of Nafion (DuPont, Wilmington, DE, USA) [69,70].

In the case of a cation-exchange membrane, the modifying film was made using the layer-by-layer method. It consisted of 4 bilayers of successively deposited aliphatic polyelectrolytes. The first aliphatic polyelectrolyte contained positively charged ammonium groups. The second aliphatic polyelectrolyte contained negatively charged sulfonic groups. An aliphatic polyelectrolyte with a high content of amino groups coated these layers. Membranes with a modified surface are designated by the index *m*.

## 3. Results and Discussion

### 3.1. ED Processing of the Model Solution

**Figure 2:**
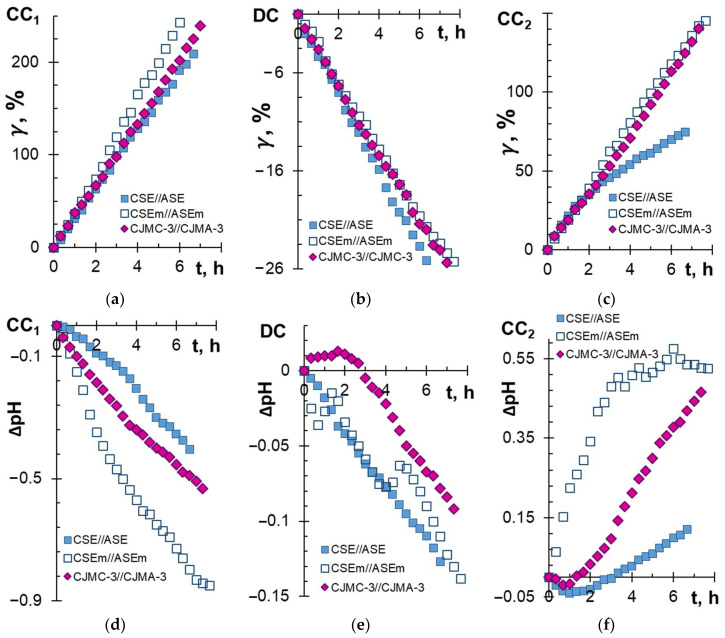
Degrees of concentration (**a**,**c**) and demineralization (**b**) of solutions, as well as the difference between the current and initial pH values (**d**–**f**) in the CC_1_ (**a**,**d**), DC (**b**,**e**) and CC_2_ (**c**,**f**) circuits vs. the ED duration of the model solution. Data are presented for CC_1_, DC and CC_2_ formed by membranes CJMC-3 and CJMA-3, CSE and ASE, and CSEm and ASEm.

**Figure 3:**
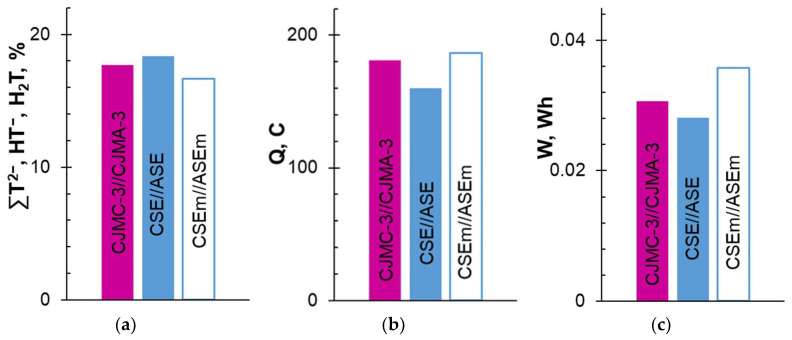
The degree of removal of tartrates (**a**), the number of electric charges transported (**b**) and energy consumption (**c**) with a decrease in the conductivity of the model solution in the desalination circuit by 20%. The data are presented for CC_1_, DC and CC_2_ formed by CJMC-3 and CJMA-3, CSE and ASE, and CSEm and ASEm membranes.

Figure 2 and Figure 3 and Table 3 present the results of electrodialysis processing of the model solution using CJMC-3 and CJMA-3, CSE and ASE, and CSEm and ASEm membranes.

With an increase in the duration of electrodialysis (ED), the conductivity of the model solution in the DC circuit expectedly decreased (Figure 2b), and the conductivity of the solutions in concentration circuits CC_1_ and CC_2_ increased (Figure 2a,c) compared with the initial values. Moreover, significant differences in the behavior of membrane stacks formed by the membrane pairs CJMC-3 and CJMA-3 and CSE and ASE were observed only for CC_2_, into which anions were transferred from the model solution. The increase in conductivity (Figure 2c) and alkalization of the solution (Figure 2f) in this circuit were more pronounced in the case of aliphatic membranes compared to aromatic membranes. These differences point to the differing composition of the anions carried by the CJMA-3 and ASE membranes. In particular, slightly more tartrates and less chloride were transported across the ASE membrane compared to CJMA-3 (Table 3). Note that the difference in recovered tartrates using aliphatic and aromatic anion-exchange membranes at a given (20%) degree of demineralization of the model solution (Figure 3b) was approximately the same.

Differences in the number of electric charges transported and energy consumption were small (Figure 3b,c). These data suggest positive prospects for the use of both studied anion-exchange membranes in the ED tartrate stabilization of wine. The same can be said about the cation-exchange membranes. Both membranes provided approximately the same increase in the conductivity of the solution in the CC_1_ circuit (Figure 2a). The difference is that the CJMC-3 membrane carried slightly more protons (Figure 2d) and slightly fewer potassium ions (Table 3) compared to the CSE membrane. The revealed differences in transport characteristics seem to be due to the higher exchange capacity of ASE and CSE compared to CJMA-3 and CJMC-3 membranes (Table 1).

Note that the degree of removal of tartrates using the studied membrane pairs (*γ_T_* = 19 ± 1%) is almost proportional to the degree of demineralization of the solution (*γ* = 20 ± 1%). Moreover, the use of membranes of the previous generation ensured the extraction of 10–15% of tartrates while reducing the conductivity of the processed solution by 20% [35], possibly as a result of the fouling stage of the CJMC-3, CJMA-3, CSE and ASE membranes, which were in contact with the wine material for only 6–8 h. Furthermore, fouling takes place and depends on the nature of the studied membranes.

### 3.2. Fouling of Membranes in Electrodialysis

#### 3.2.1. Fouling Color Indication

**Figure 4:**
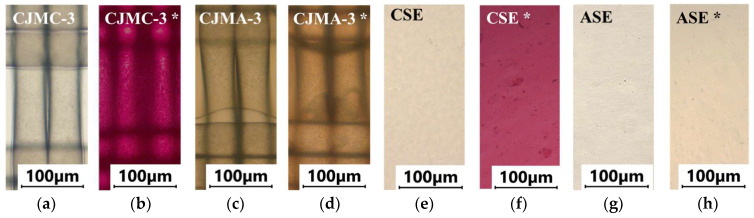
Optical images of membranes surfaces facing the desalination compartment. Membranes after electrodialysis processing of model solution (**b**,**d**,**f**,**h**) are indicated by *. Images of pristine membranes in distilled water (**a**,**c**,**e**,**g**) are shown for comparison.

**Figure 5:**
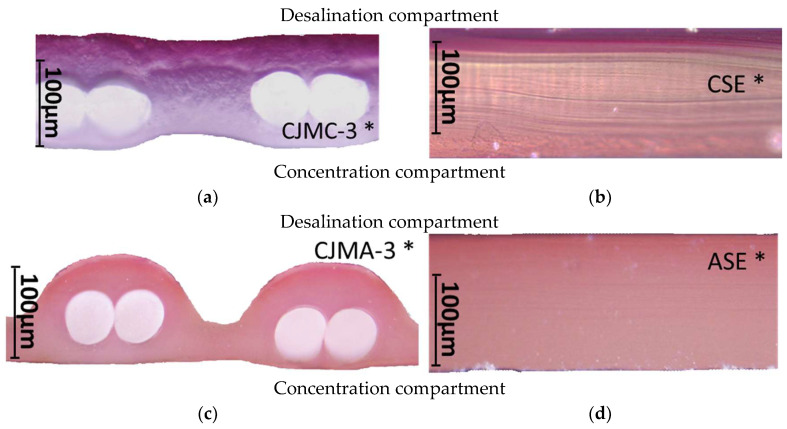
Optical images of cross sections of the studied cation-exchange (**a**,**b**) and anion-exchange (**c**,**d**) membranes after electrodialysis, indicating the location of the DC (desalination compartment) and CC_1_ or CC_2_ (concentration compartment) relative to the membrane cross sections.

Figure 4 and Figure 5 show optical images of the surfaces of the membranes facing the desalination compartments, as well as their cross sections.

The surfaces of the cation-exchange membranes acquired a burgundy (red–violet) color after ED (Figure 4b,f). The intensity of this coloration reached its maximum in a thin layer on the surface facing the DC compartment with the model solution. It decreased as it approached the opposite surface facing CC_1_ with a KCl solution. As in the case of cranberry juice [45] flavylium, anthocyanin cations (Ant^+^), which are red in an acidic environments (SM, Figure S5), penetrated into the volume of CJMC-3 * and CSE *, and high-molecular-weight proanthocyanidins (PACs), tannins Ant complexes with wine components were adsorbed on the surface. The purple shade of the CJMC-3 * cross section appears to be associated with complexes of anthocyanins with (metal) ions, which are blue in color [71,72]. These positively charged complexes penetrated deep into CJMC-3 * due to the high mobility of its polymer chains. However, the diffusion of these complexes deep into CSE * was hindered by stronger crosslinking of its aromatic polymer matrix. That is why only a thin layer near the surface facing the DS acquired a purple shade.

The surfaces of anion-exchange membranes after ED (Figure 4d,h) have a shade that is close to the color of the surfaces of pristine membranes (Figure 4c,g). However, this does not mean that anthocyanins and their complexes are absent on the surfaces and in the depths of CJMA-3 * and ASE * membranes. It is known that the pH of the internal solution of anion-exchange membranes is shifted to the alkaline region compared to the external solution due to the Donnan exclusion of protons, which are coions for AEM [73]. Therefore, at a pH of the external solution of about 3 (as in the studied model solution), the internal solution of AEM has a pH of about 6–7. With such values of pH, the red anthocyanin cations turn into a mixture of colorless carbinol pseudobases and purple quinoidal anhydrobases (SM, Figure S5), the color of which (SM, Figure S6) is similar to that of CJMA-3 * and ASE * membranes (Figure 4d,h). After the cross sections of anion-exchange membranes were placed in an acidic medium, they acquired a red shade, indicating the presence of Ants in the deep of CJMA-3 * and ASE * (Figure 5c,d). In addition, as in the case of cation-exchange membranes, a very thin layer of burgundy substances appeared on the surface of the AEMs.

The results of color indication of fouling were confirmed by IR spectroscopy data and estimates of the hydrophilic–hydrophobic balance of the membrane surface.

#### 3.2.2. IR Spectra and Surface Contact Angles of the Membranes Surfaces

**Figure 6:**
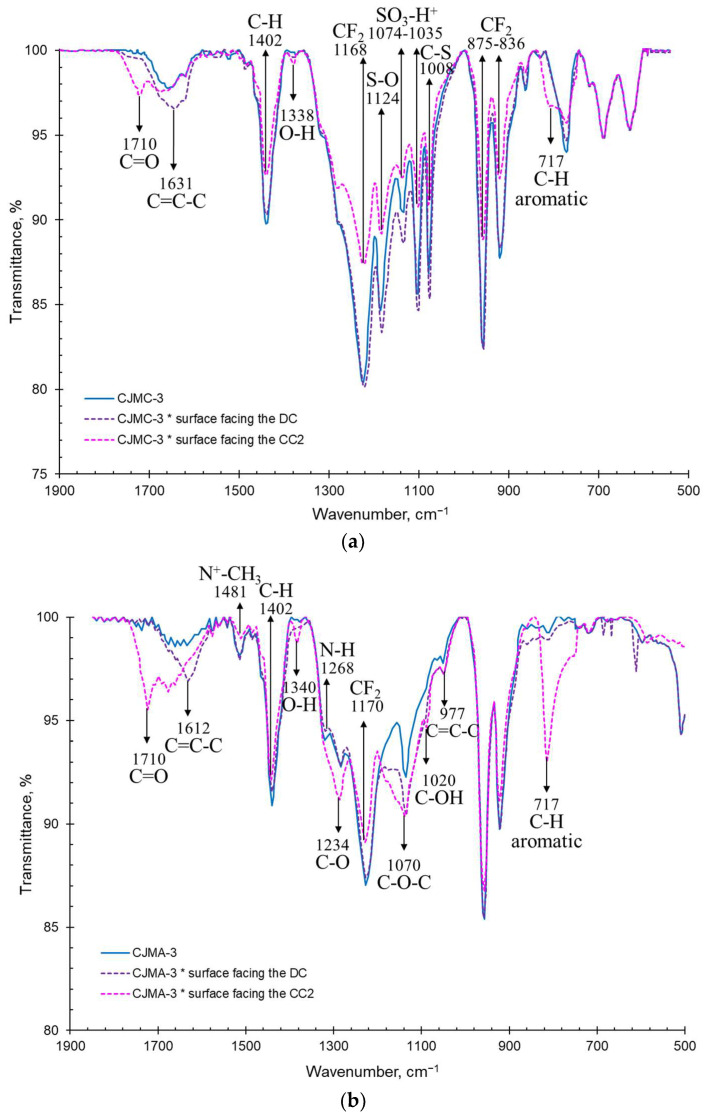
IR spectra of CJMC-3 (**a**) and CJMA-3 (**b**) membranes before and after ED.

**Figure 7:**
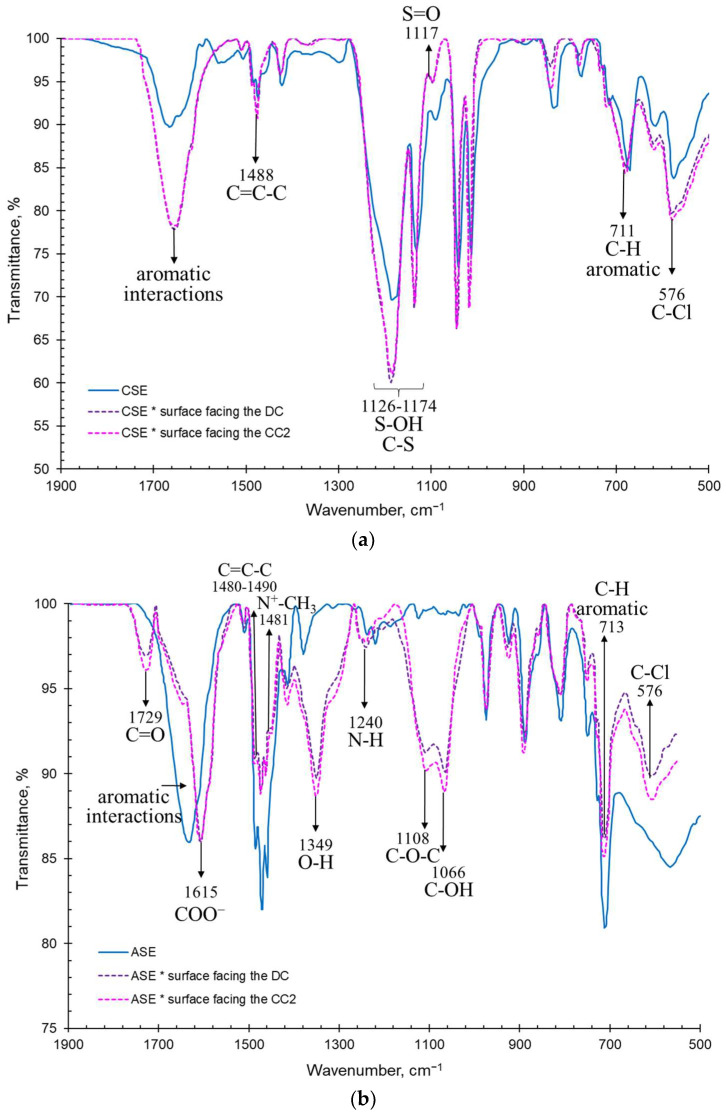
IR spectra of CSE (**a**) and ASE (**b**) membranes before and after ED.

The IR spectra of pristine and fouled membranes are shown in Figure 6 and Figure 7. The surfaces of the fouled membranes that were facing the concentration compartments are designated CC. The surfaces that were exposed to the desalination compartment with the model solution are designated as DC.

The pristine membranes CJMA-3 and CJMC-3 (Figure 6) have a fluoropolymer aliphatic matrix, which is indicated clearly by the peaks inherent to both of these membranes. The strong absorption bands at 1768 cm^−1^ and 877 cm^−1^ characterize asymmetric and symmetric vibrations of the CF_2_ bond, respectively [74]. The range of 1200–1300 cm^−1^ refers to vibrations of the C-F bond [75]. In addition, both membranes have an absorption peak in the region of approximately 1403 cm^−1^, indicating the presence of a C-H bond. Moreover, there are weakly intense bands in the regions of 837 cm^−1^, 970–980 cm^−1^ and 1610–1650 cm^−1^, which together confirm the presence of aromatic material as a crosslinking component [76,77].

Functional groups of the CJMA-3 membrane are characterized by the presence of C-N and N-CH_3_ vibrations (1481 cm^−1^ and 1232 cm^−1^, respectively), which confirms the presence of quaternary ammonium functional groups [4] and a small number of secondary amino groups (1268 cm^−1^) [78]. The most significant peaks characteristic of sulfonate groups are found in the CJMC-3 membrane, which include oscillation of C-S, S-O and SO_3_-H^+^ in the range of 1008–1126 cm^−1^ [79]. Thus, the materials from which the studied membranes are made are stable, not harmful or toxic and allowed for use in food industries [80,81].

The spectrum of the CJMC-3 * and CJMA-3 * membranes facing CC (Figure 6) shows the presence of -C=O (~1710 cm^−1^) and -OH (~1350 cm^−1^) groups, which are inherent in anthocyanins [82] and usually attributed to the galloyl units of catechins (proanthocyanidins) [79]. Screening of functional groups and groups attributed to the polymer matrix is also noticeable for both membranes. The peaks of symmetric vibrations at 700–750 cm^−1^ are related to skeletal vibrations of sugar in glycosylated phenols [83]. In the case of the CJMA-3 * membrane, the peaks in the region of 1000-1200 cm^−1^ show C-OH, C-O and C-O-C valence vibrations related to vibrations of fragments of alcohol and sugar functional groups, as well organic acids [84]. The same peaks are observed in the IR spectra of the surface facing the DC. However, the -C=0 groups (1710 cm^−1^) disappear, but C=C-C groups (1612 cm^−1^) are observed. This suggests the presence of anthocyanins and other low-molecular-weight polyphenols in the bulk of both membranes, whereas high-molecular-weight polyphenols occupy the surface facing the DC.

The IR spectra of the CSE and ASE membranes (Figure 7) contain peaks related to stretching vibrations of the C-H (~3030 cm^−1^) and aromatic ring (1440–1600 cm^−1^), as well as in-plane (960–1000 cm^−1^) and out-of-plane deformation vibrations of the benzene ring (675–950 cm^−1^), which clearly demonstrate the aromatic nature of their matrix [75]. In particular, strong absorption bands in the “fingerprint” region characteristic of the benzene ring, as well as peaks at 1460–1495 cm^−1^ and 1635 cm^−1^, are characteristic of a polystyrene-divinylbenzene matrix [77]. The spectra of both membranes exhibit peaks characteristic of the C–Cl groups (576 cm^−1^) [85]. Inert fillers (polyvinyl chloride or other chlorinated aliphatic polymers) may contain these groups. In the ASE membrane, the transmission band of carbon–nitrogen bonds (1120 cm^−1^ and 1470 cm^−1^) are an indirect sign of quaternary amines. A weakly pronounced peak at 1240 cm^−1^ indicates the probability of the presence of a small number of secondary ammonium groups. The CSE cation-exchange membrane shows distinct peaks of C-S, S=O and O-S=O in the range of 1008–1126 cm^−1^ [79] corresponding to the -SO_3_^-^ groups. The shift of peaks relative to each other in the membranes can be related to compression stretching and different strains of matrices with different functional groups for CSE and ASE.

Everything that has been discussed for aliphatic membranes after electrodialysis is true for aromatic membranes. In addition, a strong increase in the transmittance of peaks related to the aromatic components of wine is observed through their π–π (stacking) interaction with the CSE and ASE aromatic matrix [85]. The spectrum of the ASE * membrane (Figure 7b) acquires a pronounced peak at approximately1600 cm^−1^, which is attributed to the carboxyl group COO^−^ [84]. The anions of organic (in particular tartaric) acid are counterions for anion-exchange membranes. Therefore, they are present in the bulk of the membrane. This peak becomes clearly visible due to the higher ion-exchange capacity of the ASE compared to the CJMA-3 membrane (Table 1). An increase in the peak of 1240 cm^−1^ for the ASE * membrane indicates the adsorption of substances that contain secondary amines, such as proteins and amino acids.

The contact angle values (Table 4) makes it possible to estimate the hydrophilic/hydrophobic balance of the pristine membrane surface and its changes after ED. Wet CJMC-3 and CJMA-3 membranes demonstrate rather low wetting angles. This result is not expected because the ion-exchange capacity of aliphatic membranes is relatively low (Table 1). Therefore, their surface should have a low concentration of hydrophilic polar groups. In addition, hydrophobic fluorine-containing fragments of the matrix should contribute to an increase in contact angles. The observed hydrophilicity of the CJMC-3 and CJMA-3 surface was caused by the large macropores localized at the boundary between the ion-exchange material and the reinforcing cloth that peers through to the surface (Figure 5a) [62,63,68]. Judging by the ion-exchange capacity (Table 1), the concentration of polar fixed groups on the surface of CSE and ASE membranes is approximately three times higher compared to CJMC-3 and CJMA-3. Moreover, the contact angles of the surfaces of aromatic membranes are higher than in the case of aliphatic membranes (Table 4). The obtained data are most likely explained by the fact that CSE and ASE membranes do not contain reinforcing cloth (Figure 5b,d) or, accordingly, macropores.

Adsorption of highly hydrated complexes of wine components leads to significant hydrophilization of the membrane surface (Table 4).

Thus, the results of IR spectroscopy and assessment of the hydrophilic–hydrophobic balance of surfaces confirmed and detail the conclusions made on the basis of the color indication of the studied membranes. The low-molecular-weight components of wine (primarily anthocyanins and catechins) penetrated deep into all studied membranes. However, during the initial stage of fouling (as in our studies), they do not cause irreparable harm to functional groups, despite the fact that they partially shield them. High-molecular-weight polyphenols, as well as their hydrophilic complexes with metal ions, saccharides, amino acids, etc., are adsorbed on the surfaces of membranes facing the DC. The structural features of the membrane matrix are manifested as follows. In the case of aromatic membranes, fouling is facilitated by π-π (stacking) interactions the ion-exchange matrix and polyphenols. Fouling of the volume of aliphatic membranes is facilitated by the higher mobility of their polymer chains.

The blue complexes of Ant with counterions of Mg^2+^, K^+^ and other metals [71] are observed in the depths of the CJMC-3 * cation-exchange membrane. Stronger crosslinking of the ion-exchange matrix of the CSE aromatic membrane hinders the penetration of these complexes into its depth.

#### 3.2.3. Current–Voltage Curves and Electrical Conductivity of Membranes

**Figure 8:**
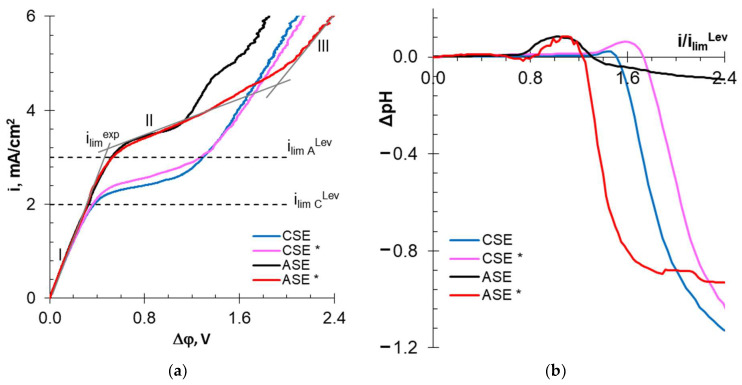
Current–voltage curves (**a**) of aromatic membranes before (CSE and ASE) and after (CSE * and ASE *) electrodialysis processing of the model solution, as well as the dependence of the solution pH difference at the DC output and input upon the current density normalized to the calculated limiting current (**b**). The dependences of ΔpH vs. *i/i_lim_^Lev^* were obtained in parallel with the CVC measurements. The studies were carried out in 0.02 M NaCl solution.

**Figure 9:**
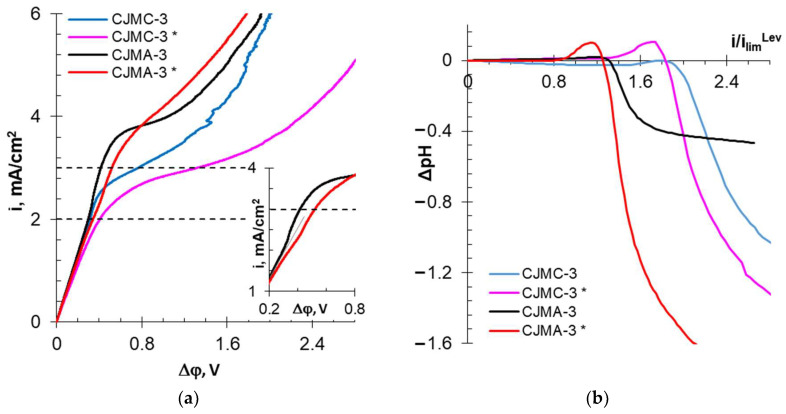
Current–voltage curves (**a**) of aromatic membranes before (CJMC-3 and CJMA-3) and after (CJMC-3 * and CJMA-3 *) electrodialysis processing of the model solution, as well as the dependence of the solution pH difference at the DC output and input upon the current density normalized to the calculated limiting current (**b**). The dependences of ΔpH vs. *i/i_lim_^Lev^* were obtained in parallel with the CVC measurements. The studies were carried out in 0.02 M NaCl solution.

Figure 8 and Figure 9 show the current–voltage curves (CVCs) and the dependences of the solution pH difference at the output and input DC upon the current density normalized to the calculated limiting current. These dependences provide information abouts the influence of membrane surface characteristics on the development of water splitting and electroconvection.

CVCs of the pristine membranes are similar to many others obtained, for example, for CMX or AMX [86,87]. The curves contain the initial (I) and “overlimiting” (III) sections, as well as the “sloping plateau” section (III). The slopes of section (I) are approximately the same for all the studied membranes, as their electrical resistance is small compared to the resistance of the solution located between the Luggin capillaries. The intersection point of the tangents to sections I and II gives the value of the experimental limiting current (*i_lim_^exp^*). The difference in the values of the potential drops at the intersection points of the tangents to sections II and III and I and II gives the value of the “plateau length”. These parameters are summarized in Table 5.

All pristine membranes show *i_lim_^exp^* exceeding theoretical limiting currents. Additionally, as in the case of well-studied membranes, for example, AMX [88], the reason for this excess is the development of electroconvection by the “electroosmosis I” mechanism [89]. This phenomenon manifests itself as an increase in conductivity in section I at currents preceding *i_lim_^exp^* compared to the conductivity during the early stages of concentration polarization (inset in Figure 9a). The decisive role for the development of “electroosmosis I” on the surface of the membranes under study seems to be played by the geometric inhomogeneity (waviness) of their surface. Indeed, CJMC-3 and CJMA-3 have a wavier surface compared to CSE and ASE. Therefore, the conductivity of the solution in the end of section I increases more significantly, and the increase in *i_lim_^exp^* compared to *i_lim_^Lev^* is higher than in the case of CSE and ASE membranes.

In the case of homogeneous ion-exchange membranes, the start of water splitting, as a rule, coincides with the achievement of *i_lim_^exp^* [86]. The studied pristine membranes are no exception. As follows from the results of IR spectroscopy, CJMA-3 and ASE contain a certain amount of secondary and tertiary amines, which exhibit highly catalytic activity towards water splitting [90]. The pristine CJMA-3 membrane contains more weakly basic fixed groups than pristine ASE. This conclusion is based on the more intense acidification of the desalted solution in the case of the CJMA-3 membrane compared to ASE.

Sulfonate fixed groups have low catalytic activity with respect to water splitting [90]. In addition, “electroosmosis I” shifts the start of water splitting to 1.35 (CSE) and 1.50 (CJMC-3) *i/i_lim_^Lev^* (Table 5). However, alkalization of the desalted solution, which indicates a more intense generation of H+ and OH- ions at the CEM/depleted solution interface, can be detected only with a slight excess of *i_lim_^exp^*. At higher current densities, water splitting near the surface of the MA-41 auxiliary membrane (which is used to measure the CVCs of cation-exchange membranes) “wins”. Therefore, the desalted solution is acidified in overlimiting current modes. Less acidification of the solution in the case of CJMC-3 compared to CSE is an indicator of more intense water splitting on the surface of the aliphatic membrane compared to the aromatic membrane.

In the case of cation-exchange membranes, the “plateau length” causes a critical potential drop, at which point large vortex structures arise (Rubinstein–Zaltzman non-equilibrium electroconvection [91]) that intensively mix the solution and enhance mass transfer. The hydrophilicity of the surface the two studied CEM membranes is almost the same. However, slightly more intense water splitting apparently shifts the formation of large electroconvective clusters to the region of higher potential drops. Therefore, the plateau on the CVC of the CJMC-3 membrane is lengthened compared to the CSE (Table 5). In the case of anion-exchange membranes, the “plateau length” decreases due to the starting of intense water splitting.

After ED processing of the model solution, the values of the experimental limiting currents changed little in comparison with pristine membranes. The CVC of the aromatic CSE * membrane underwent the fewest changes (Figure 8a, Table 5); *i_lim_^exp^* increased by only 7%, and the “plateau length” remained the same as for the pristine membrane. However, less acidification of the solution (Figure 8b and Table 5) indicates a slight enhancement of water splitting in the case of CSE * compared to the CSE membrane. This means that the above-described fouling with wine components did not lead to dramatic changes in the electrochemical characteristics of the CSE.

In the case of CJMC-3 *, the plateau length increased by 1.5 times, and desalted solution become acidic in comparison with the CJMC-3 membrane (Figure 9, Table 5). This means that the foulant on the CJMC-3 * surface reduced the ability of the membrane to water split. However, significant hydrophilization of the surface (Table 4) due to this foulant hindered the onset of intense electroconvection.

The CVC plateau of the ASE * and CJMA-3 * membranes lengthened by more than 200 and 15%, respectively. The desalted solution became significantly acidified compared to the pristine membranes (Figure 8 and Figure 9 and Table 5). This means that the hydrophilization of the surface caused by the adsorption of wine components (Table 4) contributed to a reduction in electroconvection. Specific interactions of anions of tartrates and other polybasic acids with fixed groups of the anion-exchange membrane cause a change in the positive electric charge to a negative charge [92]. Both hydrophilization and surface recharge inhibit electroosmosis I. As a result, the section of negative conductivity on the CVC practically disappears (insert in Figure 9a). Small vortices developing by the mechanism of “electroosmosis I” begin to rotate in the opposite direction and prevent the development of non-equilibrium electroconvection [93]. It is known [90] that carboxylic groups have exhibit highly catalytic activity towards water splitting. In addition, positively charged fixed groups on the AEM surface can form a bipolar interface with an adsorbed foulant layer that contains complexes or colloidal particles with negatively charged carboxyl groups.

Thus, fouling of the surface of aliphatic and aromatic membranes leads to different consequences. In the case of cation-exchange membranes, water splitting increases on the surface of the aromatic CSE * membrane but decreases on the surface of the aliphatic CJMC-3 * membrane. In contrast, in the case of anion-exchange membranes, water splitting increases on the surface of the aliphatic CJMA-3 *a membrane but decreases on the surface of the aromatic CSE * membrane. It is quite probable that the reason for the observed phenomenon is the change in the surface charge of the anion-exchange membranes due to fouling. However, this hypothesis requires further confirmation.

Conductivity (Table 6) is largely governed by the transport characteristics of the membrane volume.

Short-term (6–8 h) participation in ED processing of the model solution has practically no effect on the conductivity of the aliphatic CJMC-3 * cation-exchange membrane. Moreover, the conductivity of the CJMA-3 * membrane decreases by about 25 ± 4%. In the case of both aromatic membranes, fouling causes a decrease in conductivity by 16 ± 4% (ASE *) and 28 ± 4% (CSE *). In the case of aromatic membranes, the presence of a dense layer of foulants under the surface facing the DC (Figure 5) seems to play a decisive role. The decrease in the conductivity of the aliphatic CJMA-3 * is most likely caused by specific interactions of fixed amino groups with carboxyl groups of anions of multiply charged organic acids, which are transferred through the anion-exchange membrane during ED of the model solution.

### 3.3. Impact of Membrane Modification on the Fouling

Modifying the surfaces of CSE and ASE membranes should have resulted in repla-cement of the aromatic surface with an aliphatic surface, as well as a reversal of its electrical charge. In the case of the modified cation-exchange membrane, CSEm, the surface acquired the same positive charge as that of the Ant^+^ cations in the model solution (and wine materials). In the case of the modified anion-exchange membrane, ASEm, the surface acquired the same negative electric charge as that of the counterions and the colloidal particles of wine component complexes [94] on the AEM surface.

The CSEm surface became more hydrophobic, whereas the ASEm surface became more hydrophilic compared to the pristine membranes (Table 4). These changes in hydrophilic/hydrophobic balance appear to be caused by a decrease in the polar groups on the CSEm surface compared to CSE. It is known that films formed using LF-4SK (or Nafion) dispersion have a porous structure and a rather hydrophilic surface. High hydrophobicity of surfaces is achieved by the treatment of samples at high temperatures [95]. The ASEm membrane was not temperature-treated due to fears of destroying its structure.

The conductivity of ASEm and ASE was approximately the same within measurement error (Table 6). The conductivity of CSEm decreased by four times compared to the pristine membrane (Table 6). These changes in conductivity are in agreement with the observed changes with similar surface modifications of other membranes [96,97]. Indeed, a thin single layer of LF-4SK does not significantly contribute to the resistance of counterion transfer in the anion-exchange membrane. On the contrary, the formation of several dense bilayers with fixed groups of different charges can significantly increase this resistance [96,98] if the thickness of each layer is more than a few nanometers, as in our case.

The time it took to reduce the conductivity of the model solution increased by 21 ± 1% compared to the ED processing of the model solution using CSE and ASE membranes (Figure 2b). The pH of the model solution decreased by only 0.14 units (Figure 2e), which is allowed by the requirements for tartrate stabilization of wine [99]. The degree of tartrate removal using the modified membranes slightly decreased compared with pristine membranes (Figure 3a). The flux of tartrates through ASEm slightly reduced compared to ASE (Table 3). However, the transport of Cl^−^ anions through ASEm and K^+^ cations through CSEm increased compared to pristine membranes (Table 3). The reason for these changes was the competitive transfer of protons (CSEm) and hydroxyl ions (ASEm) due to water splitting at the CSE/m and ASE/m interfaces (m is a modifier). More significant acidification and alkalization of the solutions in circuits CC_1_ and CC_2_ (Figure 2d,f) confirm this assumption. In addition, the transferred electrical charge and energy consumption increased by 17% and 29%, respectively, when replacing CSE and ASE membranes with CSEm and ASEm (Figure 3b,c). The deterioration of the energy performance of ED processing of the model solution was influenced by an increase in the electrical resistance of the modified membranes.

**Figure 10:**
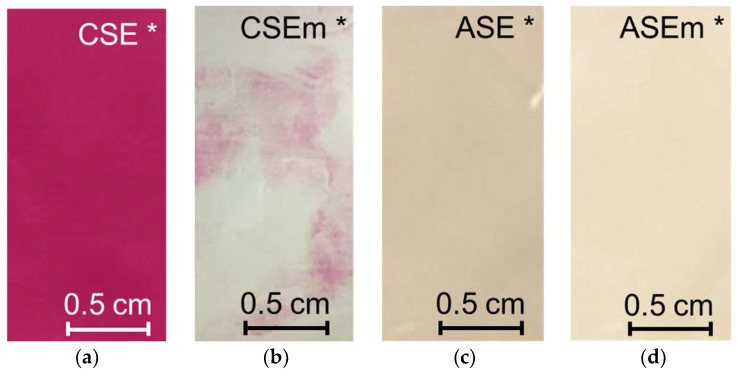
Optical images of the surfaces of CSE and ASE (**a**,**c**) and modified CSEm and ASEm (**b**,**d**) membranes facing the desalination compartment. The membranes after electrodialysis processing of the model solution are indicated by *.

**Figure 11:**
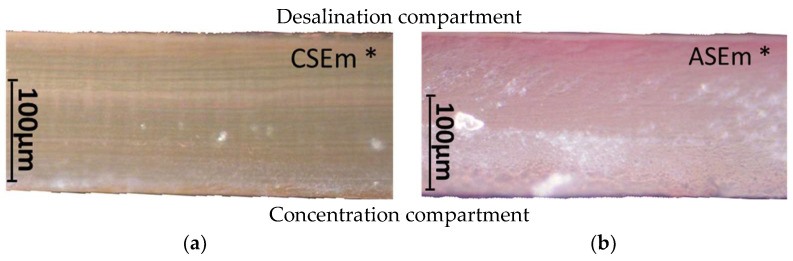
Optical images of cross sections of modified CSEm * (**a**) and ASEm * membranes (**b**) after ED processing of model solution. “Desalination compartment” and “Concentration compartment” indicate the chambers bordering the membrane surface. The cross section of the ASEm * membrane was placed in acidic solution before imaging to make the presence of anthocyanins more evident.

Figure 10 and Figure 11 visualize the fouling of modified membranes by anthocyanins and other colored wine components. Table 4 contains information on the effect of foulants on the hydrophilic/hydrophobic balance of the CSEm and ASEm membrane surfaces.

After ED, a decrease in the contact angle value by 28% (Table 4) (increase in hydrophilicity) of the surface was observed in the case of CSEm * membrane. However, the CSEm * surface remained more hydrophobic than in the case of the CSE * membrane (Table 4). Only some islands on the surface turned pink, whereas the entire surface of the unmodified membrane was bright red (Figure 10a,b, Table 4). Electrodialysis of the model solution did not affect the hydrophilic/hydrophobic balance of the surface of the modified ASE membrane (Table 4). The surface of the ASEm * became lighter compared to the ASE * membrane (Figure 10c,d). Color indication of the cross sections (Figure 11) showed significantly less fouling of the modified membranes compared to the pristine membranes (Figure 5b,d). The reduction in fouling of ASEm * volume was less significant compared to the cation-exchange CSEm * membrane.

Thus, modification of the surface of aromatic membranes protects their volume from anthocyanins and other coloring components of wine. Moreover, the conductivity of the modified CSEm and ASEm after ED decreased more strongly compared to the pristine membranes (Table 6). The reason for the decrease in the conductivity may be a more intense transfer of protons (CSEm) or hydroxyl ions (ASEm) due to the enhancement of water splitting during ED. For example, increasing the alkalinity of the internal solution of the ASEm membrane will unequivocally lead to the transfer of mainly doubly charged tartrate anions [100]. As previously mentioned, such anions enter into specific interactions with fixed groups of AEMs, reducing their exchange capacity [101] and, accordingly, their conductivity [102].

Thus, the results of our first attempt to counteract fouling in the tartrate stabilization of red wine using electrodialysis confirmed the promise of modifying the surface of aromatic ion-exchange membranes. Moreover, the use of the layer-by-layer method was more effective than applying a single layer of modifier. However, preventing the penetration of Ant and PACs into ion-exchange membranes slightly increased the duration of ED and energy consumption. The optimization of the parameters of the modifying layers will be continued in the future.

## 4. Conclusions

CSE and ASE membranes contain an aromatic ion-exchange matrix and an apparently inert filler from an aliphatic chlorine-containing material. CJMC-3 and CJMA-3 membranes have an aliphatic polyvinylidene fluoride ion-exchange matrix crosslinked with aromatic agents.

The wavier surface of aliphatic membranes stimulates more intense electroconvection in the desalination compartment compared to aromatic membranes. The CJMA-3 anion-exchange membrane contains more weakly basic fixed groups and therefore shows increased water splitting compared to the ASE membrane.

Under the identical conditions of ED processing of the model solution of a red wine (current density 1.22 mA/cm^2^ and average flow rate 0.42 cm/s), the studied membrane pairs provided a decrease in the electrical conductivity of wine by 20 ± 1% at an energy consumption of 31 10^−3^ Wh (CJMC-3//CJMA-3) and 28 10^−3^ Wh (CSE//ASE). The degree of removal of tartrates was 18 ± 1% and 20 ± 1%, respectively.

After a short-term (6–8 h) ED treatment of the model solution, fouling with highly hydrated wine components led to significant hydrophilization of the surface of all the studied membranes.

The high-molecular-weight aromatic components of red wine are localized in the surface facing the desalination compartment, as well as in the near-surface layer of CSE and ASE membranes. Anthocyanins and other colored wine components occupy the entire volume of aliphatic membranes after electrodialysis. This indicates a less dense crosslinking of the CJMC-3 and CJMA-3 membranes compared to the studied aromatic membranes.

Fouling of the surface of the aliphatic and aromatic membranes leads to different consequences. In the case of cation-exchange membranes, water splitting increases on the surface of the aromatic CSE membrane but decreases on the surface of the aliphatic CJMC-3 membrane. In contrast, in the case of anion-exchange membranes, water splitting increases on the surface of the aliphatic CJMA-3 a membrane but decreases on the surface of the aromatic CSE membrane. This phenomenon can be explained by a change in the surface charge of anion-exchange membranes due to specific interactions with carboxyl groups of anions of polybasic acids. However, this hypothesis requires further confirmation.

The casting of aliphatic polyelectrolyte films protects the surface and volume of the studied aromatic membranes from anthocyanins and other coloring components of wine in ED. Approximately the same recovery of tartrates is achieved with a relatively small increase in energy consumption compared to using pristine CSE and ASE membranes. The layer-by-layer method of modification seems to be preferable.

## Figures and Tables

**Table 1:** Some characteristics of the studied membranes.

Membrane	Thickness in 0.02 M NaCl Solution (µm)	Water Content (gH_2_O/g_dry_, %)	Ion-Exchange Capacity of Swollen Membrane (mmol/g_wet_)
CJMA-3	151 ± 5	17 ± 1 [62]	0.57 ± 0.05 [62]
CJMC-3	185 ± 5	44 ± 3 [63]	0.63 ± 0.05 [63]
ASE	150 ± 5	24 ± 1 [56]	1.93 ± 0.05 [56]
CSE	140 ± 5	42 ± 1 [56]	1.57 ± 0.05 [56]

**Table 2:** Detailed composition of the wine.

Inorganic Matter	Concentration		Organic Matter	Concentration	
	mg/L	%mass		mg/L	%vol
Cl^−^	193 ± 5	–	acetic aldehyde	32 ± 5	–
SO_4_^2−^	969 ± 5	–	diacetyl	4 ± 5	–
K^+^	1281 ± 5	–	acetoin	48 ± 5	–
Na^+^	136 ± 5	–	furfurol	14 ± 5	–
Mg^2+^	110 ± 5	–	2,3-butyleneglycol racemate	560 ± 5	–
Ca^2+^	124 ± 5	–	2,3-butyleneglycol meso	259 ± 5	–
**Organic acids**			methyl acetat	45 ± 5	–
ascorbic	31 ± 5	–	ethyl acetat	82 ± 5	–
tartaric	1872 ± 5	–	ethyl butyrate	1 ± 5	–
citric	405 ± 5	–	ethyl caproate	7 ± 5	–
lactic	5020 ± 5	–	ethyl lactate	83 ± 5	–
malic	156 ± 5	–	ethyl caprylate	2 ± 5	–
succinic	1622 ± 5	–	ethyl caprate	4 ± 5	–
chlorogenic	11 ± 5	–	ethanol	–	11 ± 1
niacin	8 ± 5	–	methanol	142 ± 5	–
orotic	973 ± 5	–	1-propanol	33 ± 5	–
caffeic	37 ± 5	–	isobutanol	50 ± 5	–
gallic	10 ± 5	–	1-butanol	2 ± 5	–
acetic	1955 ± 5	–	amyl alcohol	253 ± 5	–
propionic	9 ± 5	–	octanol	16 ± 5	–
isobutyric	1 ± 5	–	benzyl alcohol	15 ± 5	–
3-methylbutanoic	7 ± 5	–	phenethyl alcohol	128 ± 5	–
**Saccharides**			1,2-propylene glycol	24 ± 5	–
** total	–	10 ± 1	* polyphenols, total	670 ± 10	–

* total amount of monomers and polymers in terms of gallic acid; ** data provided by the manufacturer.

**Table 3:** Some parameters of electrodialysis processing of model solution at a degree of demineralization equal to 20%.

Membrane	Cation-Exchange Membrane				Anion-Exchange Membrane					
	H^+^	K^+^			Cl^−^			∑T		
	*j_i_*	^1,2^ mmol	*γ_i_*, %	^3^ *j_i_*	mmol	*γ_i_*, %	*j_i_*	mmol	*γ_i_*, %	*j_i_*
CJMC-3//CJMA-3	0.010 ± 0.002	1.09	19 ± 1	0.07	0.29	18 ± 1	0.02	0.70	18 ± 1	0.05
CSE//ASE	0.012 ± 0.002	1.19	21 ± 1	0.09	0.30	18 ± 1	0.02	0.77	20 ± 1	0.06
CSEm//ASEm	0.018 ± 0.002	1.20	21 ± 1	0.08	0.58	35 ± 1	0.04	0.65	17 ± 1	0.04

^1^ the amount of substance (in mmol) removed from the DC with a decrease in the conductivity of the model solution by 20%; ^2^ the error in determining the amount of the substance removed from the DC is ±0.002; ^3^ the error in determining the flux (*j_i,_* in mmol m^−2^ s^−1^) of ions through the membranes of the DC is ±0.02.

**Table 4:** Contact angles of the surface of the studied membranes.

Membranes	Pristine	* After ED
CJMC-3	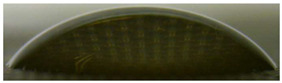	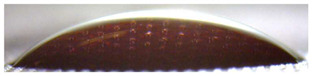
	50 ± 3	36 ± 3
CJMA-3	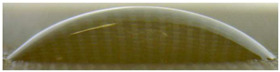	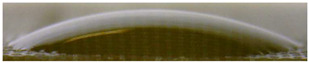
	48 ± 3	19 ± 5
CSE	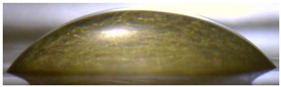	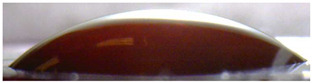
	53 ± 2	36 ± 3
ASE	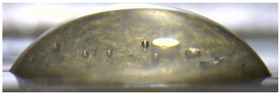	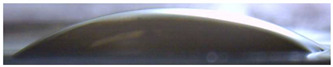
	57 ± 3	30 ± 3
CSEm	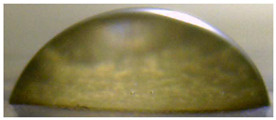	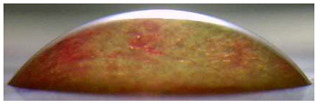
	67 ± 4	48 ± 3
ASEm	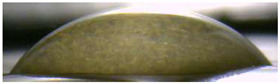	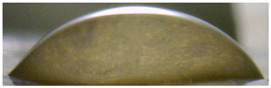
	51 ± 3	52 ± 3

* Surface facing the desalination compartment.

**Table 5:** Parameters of the current–voltage curves of the studied membranes before and after electrodialysis processing of the model solution.

Parameter		CJMC-3	CJMA-3	CSE	ASE
*i_lim_^exp^*, mA/cm^2^	Pristine	2.62	3.64	2.18	3.22
	After ED	2.52	3.48	2.34	3.15
“plateau length”, V	Pristine	1.11	0.70	0.95	0.61
	After ED	1.71	0.83	0.98	1.36
*i/i_lim_^Lev^* value corresponding to the beginning of water splitting	Pristine	1.50	1.25	1.35	1.06
	After ED	1.39	1.18	1.35	1.11
ΔpH value at *i/i_lim_^Lev^* = 2.0	Pristine	–0.09	–0.42	–0.88	–0.08
	After ED	–0.42	–1.54	–0.53	–0.88

**Table 6:** Conductivity (mS/cm) of the studied membranes before and after electrodialysis processing of the model solution. Data obtained in 0.1 M NaCl solution.

Membrane	CJMC-3	CJMA-3	CSE	ASE	CSEm	ASEm
Pristine	5.7 ± 0.3	3.1 ± 0.3	8.0 ± 0.3	4.9 ± 0.3	2.0 ± 0.3	4.6 ± 0.3
After ED	5.9 ± 0.3	2.3 ± 0.5	5.8 ± 0.3	4.1 ± 0.3	1.1 ± 0.5	1.9 ± 0.5

## Data Availability

Not applicable.

## Supplementary Materials

The following supporting information can be downloaded at: https://www.mdpi.com/article/10.3390/membranes13010084/s1, Figure S1: IR spectrum of the CJMA-3 membrane; Figure S2: IR spectrum of the CJMC-3 membrane; Figure S3: IR spectrum of the ASE membrane; Figure S4: IR spectrum of the CSE membrane; Figure S5: The structures of anthocyanins and their modifications depending on the pH of the medium; Figure S6: Effect of pH on the color of the model solution.

## References

[1] Yakovlev V.A., Stepanova A.N. (2020). Analysis and Prospects for the Development of Agribusiness: Regional Aspect. IOP Conf. Ser. Earth Environ. Sci..

[2] Wollan D. (2010). Membrane and Other Techniques for the Management of Wine Composition. Managing Wine Quality.

[3] Poirier D., Bennasar M., Tarodo de La Fuente B., Gillot J., Garcera D. (1984). Clarification et Stabilisation Des Vins Par Ultrafiltration Tangentielle Sur Membranes Minérales. Lait.

[4] El Rayess Y., Mietton-Peuchot M. (2016). Membrane Technologies in Wine Industry: An Overview. Crit. Rev. Food Sci. Nutr..

[5] Tamime A.Y. (2013). Membrane Processing: Dairy and Beverage Applications.

[6] Anderson K., Nelgen S. (2011). Global Wine Markets, 1961 to 2009: A Statistical Compendium.

[7] Thoukis G. (1974). Chemistry of Wine Stabilization: A Review. Chemistry of Winemaking.

[8] Gnilomedova N., Anikina N., Vesyutova A., Oleinikova V., Gavrish V., Chayka T. (2022). Identifying Tartrate Salt Crystals in Wine Sediment. Food Process. Tech. Technol..

[9] Low L.L., O’Neill B., Ford C., Godden J., Gishen M., Colby C. (2008). Economic Evaluation of Alternative Technologies for Tartrate Stabilisation of Wines. Int. J. Food Sci. Technol..

[10] Jackson R.S. (2008). Wine Science: Principles and Applications.

[11] Cabrita M.J., Garcia R., Catarino S., Jordão A.M., Cosme F. (2016). Recent Developments in Wine Tartaric Stabilization.

[12] Filipe-Ribeiro L., Milheiro J., Guise R., Vilamarim R., Fraga J.B., Martins-Gomes C., Nunes F.M., Cosme F. (2021). Efficiency of Carboxymethylcellulose in Red Wine Tartaric Stability: Effect on Wine Phenolic Composition, Chromatic Characteristics and Colouring Matter Stability. Food Chem..

[13] Bosso A., Motta S., Panero L., Lucini S., Guaita M. (2020). Use of Potassium Polyaspartate for Stabilization of Potassium Bitartrate in Wines: Influence on Colloidal Stability and Interactions with Other Additives and Enological Practices. J. Food Sci..

[14] Ortega-Heras M., González-SanJosé M.L. (2016). Mannoproteins and Enology: Tartrate and Protein Stabilization. Recent Advances in Wine Stabilization and Conservation Technologies.

[15] Lankhorst P.P., Voogt B., Tuinier R., Lefol B., Pellerin P., Virone C. (2017). Prevention of Tartrate Crystallization in Wine by Hydrocolloids: The Mechanism Studied by Dynamic Light Scattering. J. Agric. Food Chem..

[16] Lasanta C., Gomez Benitez J. (2012). Tartrate Stabilization of Wines. Trends Food Sci. Technol..

[17] Ponce F., Mirabal-Gallardo Y., Versari A., Laurie V. (2018). The Use of Cation Exchange Resins in Wines: Effects on PH, Tartrate Stability, and Metal Content. Cienc. Investig. Agrar..

[18] Houldsworth D.W. (1980). Demineralization of Whey by Means of Ion Exchange and Electrodialysis. Int. J. Dairy Technol..

[19] Purolite FDA Conditioning of Ion Exchange Resin before Food Use. https://www.purolite.com/index/core-technologies/industry/food-and-beverage/sweetener-applications/corn-sweetener-refining-with-ion-exchange-resins/fda-conditioning-before-use.

[20] Fernandes C., Dos Santos P.C., De Pinho M.N. Wine Tartaric Stabilization by Electrodialysis. Proceedings of the CHISA 2006—17th International Congress of Chemical and Process Engineering.

[21] Claus H., Tenzer S., Sobe M., Schlander M., König H., Fröhlich J. (2014). Effect of Carboxymethyl Cellulose on Tartrate Salt, Protein and Colour Stability of Red Wine. Aust. J. Grape Wine Res..

[22] Moutounet M., Bouissou D., Escudier J.L. Effets de Traitement de Stabilisation Tartrique de Vins Rouges Par Une Gomme de Cellulose (Carboxymethylcellulose). https://www.infowine.com/intranet/libretti/libretto8096-01-1.pdf.

[23] Fischler F. Commission Regulation (EC) No 2053/97 of 20 October 1997 Amending Regulation (EEC) No 3220/90 Laying down Conditions for the Use of Certain Oenological Practices Provided for in Council Regulaiton (EEC) No 822/87. https://www.efta.int/eea-lex/31997R2053.

[24] Oenodia The 2022 Innovation. https://eurodia.com/en/linnovation-2022-smart-by-oenodia-une-nouvelle-etoile-est-nee/.

[25] Straits Research Electrodialysis Equipment Market. https://straitsresearch.com/report/electrodialysis-equipment-market.

[26] Transfert I. Eurodia/Oenodia: Eco-Friendly Membrane Processes. https://www.inrae-transfert.fr/en/107-bioprocedes-biotechnologies-blanches/327-eurodia-oenodia-eco-friendly-membrane-processes.

[27] Audinos R., Roson J.P., Jouret C. (1979). Application of Electrodialysis to the Elimination of Certain Grape Juice and Wine Components. Connaiss. Vigne Vin.

[28] Paronetto L., Paronetto L., Braido A. (1977). Some Tests on Tartrate Stabilization of Musts and Wines by Electrodialysis. Vignevini.

[29] Wucherpfennig K., Krueger R. (1975). Stabilization of Grape Juice and Wine against Tartar by Means of Electrodialysis. Membrane Ion-Exchange Freeze-Cone. Food Industry, Proceedings of the International Symposium on Separation Processes, Paris, France, 5–9 January 1975.

[30] Escudier J.L., Moutounet M., Saint-Pierre B., Battle J.L. Stabilisation Tartrique Des Vins Par Membranes: Resultats et Developments Technologiques. Proceedings of the 11 ème Colloque Viticole et Oenologique.

[31] Gonçalves F., Fernandes C., Cameira dos Santos P., de Pinho M.N. (2003). Wine Tartaric Stabilization by Electrodialysis and Its Assessment by the Saturation Temperature. J. Food Eng..

[32] El Rayess Y., Achcouty S., Ghanem C., Rizk Z., Nehme N. (2016). Clarification and Stabilization of Wines Using Membrane Processes. Recent Advances in Wine Stabilization and Conservation Technologies.

[33] Vecino X., Reig M., Gibert O., Valderrama C., Cortina J.L. (2020). Integration of Monopolar and Bipolar Electrodialysis Processes for Tartaric Acid Recovery from Residues of the Winery Industry. ACS Sustain. Chem. Eng..

[34] Soares P.A.M.H., Geraldes V., Fernandes C., dos Santos P.C., de Pinho M.N. (2009). Wine Tartaric Stabilization by Electrodialysis: Prediction of Required Deionization Degree. Am. J. Enol. Vitic..

[35] Ribéreau-Gayon P., Glories Y., Maujean A., Dubourdieu D. (2006). Handbook of Enology, Volume 2: The Chemistry of Wine—Stabilization and Treatments.

[36] Gómez Benítez J., Palacios Macías V., Szekely Gorostiaga P., Veas López R., Pérez Rodríguez L. (2003). Comparison of Electrodialysis and Cold Treatment on an Industrial Scale for Tartrate Stabilization of Sherry Wines. J. Food Eng..

[37] Mourgues J. (1993). Utilisation Des Résines Échangeuses d’ions. Rev. Oenol..

[38] Cosme F., Vilela A., Jordão A. (2017). The Role of Tartaric Acid in Grapes and Wines. Advances in Chemistry Research.

[39] Brilenok N.S., Vershinin V.I., Bakhareva M.V. (2016). Evaluation of Polyphenols Antioxidant Capacity in the Presence of Complexants by FRAP Assay. Anal. Kontrol.

[40] Pellerin G., Bazinet L., Grenier D. (2021). Effect of Cranberry Juice Deacidification on Its Antibacterial Activity against Periodontal Pathogens and Its Anti-Inflammatory Properties in an Oral Epithelial Cell Model. Food Funct..

[41] Riponi C., Nauleau F., Amati A., Arfelli G., Castellari M. (1992). Essais de Stabilisation Tartrique Des Vins Au Moyen de l’électrodialyse. Rev. Fr. D’oenol..

[42] Dammak L., Fouilloux J., Bdiri M., Larchet C., Renard E., Baklouti L., Sarapulova V., Kozmai A., Pismenskaya N. (2021). A Review on Ion-Exchange Membrane Fouling during the Electrodialysis Process in the Food Industry, Part 1: Types, Effects, Characterization Methods, Fouling Mechanisms and Interactions. Membranes.

[43] Bdiri M., Perreault V., Mikhaylin S., Larchet C., Hellal F., Bazinet L., Dammak L. (2020). Identification of Phenolic Compounds and Their Fouling Mechanisms in Ion-Exchange Membranes Used at an Industrial Scale for Wine Tartaric Stabilization by Electrodialysis. Sep. Purif. Technol..

[44] Zhao Q., Du G., Wang S., Zhao P., Cao X., Cheng C., Liu H., Xue Y., Wang X. (2023). Investigating the Role of Tartaric Acid in Wine Astringency. Food Chem..

[45] Perreault V., Sarapulova V., Tsygurina K., Pismenskaya N., Bazinet L. (2021). Understanding of Adsorption and Desorption Mechanisms of Anthocyanins and Proanthocyanidins on Heterogeneous and Homogeneous Cation-Exchange Membranes. Membranes.

[46] Mikhaylin S., Bazinet L. (2016). Fouling on Ion-Exchange Membranes: Classification, Characterization and Strategies of Prevention and Control. Adv. Colloid Interface Sci..

[47] Pismenskaya N., Bdiri M., Sarapulova V., Kozmai A., Fouilloux J., Baklouti L., Larchet C., Renard E., Dammak L. (2021). A Review on Ion-Exchange Membranes Fouling during Electrodialysis Process in Food Industry, Part 2: Influence on Transport Properties and Electrochemical Characteristics, Cleaning and Its Consequences. Membranes.

[48] Ioannidou S.M., Filippi K., Kookos I.K., Koutinas A., Ladakis D. (2022). Techno-Economic Evaluation and Life Cycle Assessment of a Biorefinery Using Winery Waste Streams for the Production of Succinic Acid and Value-Added Co-Products. Bioresour. Technol..

[49] Ncube A., Fiorentino G., Colella M., Ulgiati S. (2021). Upgrading Wineries to Biorefineries within a Circular Economy Perspective: An Italian Case Study. Sci. Total Environ..

[50] Han L. (2021). Current Strategies for the Design of Anti-Fouling Ion-Exchange Membranes.

[51] Astom Detailed Specification of IEMs Produced Astom Corporation. http://www.astom-corp.jp/en/product/10.html.

[52] Wang B., Liu F., Zhang F., Tan M., Jiang H., Liu Y., Zhang Y. (2022). Efficient Separation and Recovery of Cobalt(II) and Lithium(I) from Spent Lithium Ion Batteries (LIBs) by Polymer Inclusion Membrane Electrodialysis (PIMED). Chem. Eng. J..

[53] Zhou Y., Yan H., Wang X., Wu L., Wang Y., Xu T. (2018). Electrodialytic Concentrating Lithium Salt from Primary Resource. Desalination.

[54] Yan H., Xu C., Li W., Wang Y., Xu T. (2016). Electrodialysis To Concentrate Waste Ionic Liquids: Optimization of Operating Parameters. Ind. Eng. Chem. Res..

[55] Wang H., Yan J., Fu R., Yan H., Jiang C., Wang Y., Xu T. (2022). Bipolar Membrane Electrodialysis for Cleaner Production of Gluconic Acid: Valorization of the Regenerated Base for the Upstream Enzyme Catalysis. Ind. Eng. Chem. Res..

[56] Porozhnyy M.V., Kozmai A.E., Mareev A.A., Gil V.V. (2022). Theoretical and Experimental Study of Neutralization Dialysis of Phenylalanine–Mineral Salt Equimolar Mixture of Different Concentrations. Membr. Membr. Technol..

[57] Cao R., Shi S., Cao H., Li Y., Duan F., Li Y. (2022). Surface Composite Modification of Anion Exchange Membrane by Electrodeposition and Self-Polymerization for Improved Antifouling Performance. Colloids Surfaces A Physicochem. Eng. Asp..

[58] Yao Y., Mu J., Liao J., Dong J., Luo B., Ruan H., Shen Z., Shen J. (2022). Imparting Antibacterial Adhesion Property to Anion Exchange Membrane by Constructing Negatively Charged Functional Layer. Sep. Purif. Technol..

[59] Wang J., Liu M., Feng Z., Liu J., Liao S., Li X., Yu Y. (2023). Highly Conductive Anion Exchange Membrane with a Stable Double-Sided Anti-Fouling Structure for Electrodialysis Desalination of Protein Systems. Desalination.

[60] Wang Y., Zhang Z., Jiang C., Xu T. (2016). Recovery of Gamma-Aminobutyric Acid (GABA) from Reaction Mixtures Containing Salt by Electrodialysis. Sep. Purif. Technol..

[61] Yan H., Wang Y., Xu T. K6-5: Developing Ion Exchange Membrane for Treating High Salinity Water Using Electrodialysis. Proceedings of the 5th International Conference on Sustainable Chemical Production Process Engineering (SCPPE).

[62] Sarapulova V., Pismenskaya N., Butylskii D., Titorova V., Wang Y., Xu T., Zhang Y., Nikonenko V. (2020). Transport and Electrochemical Characteristics of CJMCED Homogeneous Cation Exchange Membranes in Sodium Chloride, Calcium Chloride, and Sodium Sulfate Solutions. Membranes.

[63] Sarapulova V., Pismenskaya N., Titorova V., Sharafan M., Wang Y., Xu T., Zhang Y., Nikonenko V. (2021). Transport Characteristics of CJMAED^TM^ Homogeneous Anion Exchange Membranes in Sodium Chloride and Sodium Sulfate Solutions. Int. J. Mol. Sci..

[64] Giusti M., Wrolstad R.E. (2005). Anthocyanins. Handbook of Food Analytical Chemistry.

[65] Newman J.S. (1973). Electrochemical Systems.

[66] Lteif R., Dammak L., Larchet C., Auclair B. (1999). Conductivité Électrique Membranaire: Étude de l’effet de La Concentration, de La Nature de l’électrolyte et de La Structure Membranaire. Eur. Polym. J..

[67] Karpenko-Jereb L., Demina O., Dvorkina G., Parshikov S., Larchet C., Auclair B., Berezina N. (2001). Comparative Study of Methods Used for the Determination of Electroconductivity of Ion-Exchange Membranes. Russ. J. Electrochem..

[68] Ponomar M., Krasnyuk E., Butylskii D., Nikonenko V., Wang Y., Jiang C., Xu T., Pismenskaya N. (2022). Sessile Drop Method: Critical Analysis and Optimization for Measuring the Contact Angle of an Ion-Exchange Membrane Surface. Membranes.

[69] Gil V., Porozhnyy M., Rybalkina O., Butylskii D., Pismenskaya N. (2020). The Development of Electroconvection at the Surface of a Heterogeneous Cation-Exchange Membrane Modified with Perfluorosulfonic Acid Polymer Film Containing Titanium Oxide. Membranes.

[70] Safronova E.Y., Yaroslavtsev A.B. (2015). Synthesis of MF-4SC Composite Membranes Exhibiting an Anisotropic Distribution of Zirconia and Ion Transport Asymmetry. Pet. Chem..

[71] Hayashi K., Abe Y., Mitsui S. (1958). Blue Anthocyanin from the Flowers of Commelina, the Crystallisation and Some Properties Thereof. Proc. Jpn. Acad..

[72] Mata R., Andersen O.M., Markham K.R. (2005). Flavonoids: Chemistry, Biochemistry and Applications.

[73] Pismenskaya N., Sarapulova V., Klevtsova A., Mikhaylin S., Bazinet L. (2020). Adsorption of Anthocyanins by Cation and Anion Exchange Resins with Aromatic and Aliphatic Polymer Matrices. Int. J. Mol. Sci..

[74] Danilczuk M., Lin L., Schlick S., Hamrock S.J., Schaberg M.S. (2011). Understanding the Fingerprint Region in the Infra-Red Spectra of Perfluorinated Ionomer Membranes and Corresponding Model Compounds: Experiments and Theoretical Calculations. J. Power Sources.

[75] Bellamy L.J. (1975). The Infra-Red Spectra of Complex Molecules.

[76] Wang Y., Peng J., Li J., Zhai M. (2017). PVDF Based Ion Exchange Membrane Prepared by Radiation Grafting of Ethyl Styrenesulfonate and Sequent Hydrolysis. Radiat. Phys. Chem..

[77] Garcia-Vasquez W., Ghalloussi R., Dammak L., Larchet C., Nikonenko V., Grande D. (2014). Structure and Properties of Heterogeneous and Homogeneous Ion-Exchange Membranes Subjected to Ageing in Sodium Hypochlorite. J. Membr. Sci..

[78] Hansima M.A.C.K., Jayaweera A.T., Ketharani J., Ritigala T., Zheng L., Samarajeewa D.R., Nanayakkara K.G.N., Herath A.C., Makehelwala M., Jinadasa K.B.S.N. (2022). Characterization of Humic Substances Isolated from a Tropical Zone and Their Role in Membrane Fouling. J. Environ. Chem. Eng..

[79] Scano P. (2021). Characterization of the Medium Infrared Spectra of Polyphenols of Red and White Wines by Integrating FT IR and UV–Vis Spectral Data. LWT.

[80] Cui Z., Drioli E., Lee Y.M. (2014). Recent Progress in Fluoropolymers for Membranes. Prog. Polym. Sci..

[81] OIV International Oenological CODEX. https://www.oiv.int/index.php/standards/international-oenological-codex.

[82] Chesnokova N., Prikhodko Y., Kuznetsova A., Kushnarenko L., Gerasimova V. (2021). Anthocyanin Films in Freshness Assessment of Minced Fish. Food Process. Tech. Technol..

[83] Burattini E., Cavagna M., Dell’Anna R., Malvezzi Campeggi F., Monti F., Rossi F., Torriani S. (2008). A FTIR Microspectroscopy Study of Autolysis in Cells of the Wine Yeast Saccharomyces Cerevisiae. Vib. Spectrosc..

[84] Agatonovic-Kustrin S. (2013). The Use of Fourier Transform Infrared (FTIR) Spectroscopy and Artificial Neural Networks (ANNs) to Assess Wine Quality. Mod. Chem. Appl..

[85] Mukhamediev M.G., Bekchanov D.Z. (2019). New Anion Exchanger Based on Polyvinyl Chloride and Its Application in Industrial Water Treatment. Russ. J. Appl. Chem..

[86] Krol J. (1999). Concentration Polarization with Monopolar Ion Exchange Membranes: Current-Voltage Curves and Water Dissociation. J. Membr. Sci..

[87] Park J.-S., Choi J.-H., Woo J.-J., Moon S.-H. (2006). An Electrical Impedance Spectroscopic (EIS) Study on Transport Characteristics of Ion-Exchange Membrane Systems. J. Colloid Interface Sci..

[88] Korzhova E., Pismenskaya N., Lopatin D., Baranov O., Dammak L., Nikonenko V. (2016). Effect of Surface Hydrophobization on Chronopotentiometric Behavior of an AMX Anion-Exchange Membrane at Overlimiting Currents. J. Membr. Sci..

[89] Abu-Rjal R., Prigozhin L., Rubinstein I., Zaltzman B. (2017). Equilibrium Electro-Convective Instability in Concentration Polarization: The Effect of Non-Equal Ionic Diffusivities and Longitudinal Flow. Russ. J. Electrochem..

[90] Zabolotskii V.I., Shel’deshov N.V., Gnusin N.P. (1988). Dissociation of Water Molecules in Systems with Ion-Exchange Membranes. Russ. Chem. Rev..

[91] Rubinstein I., Zaltzman B. (2007). Electro-Convective versus Electroosmotic Instability in Concentration Polarization. Adv. Colloid Interface Sci..

[92] Zhang Y., Pinoy L., Meesschaert B., Van der Bruggen B. (2011). Separation of Small Organic Ions from Salts by Ion-Exchange Membrane in Electrodialysis. AIChE J..

[93] Titorova V.D., Moroz I.A., Mareev S.A., Pismenskaya N.D., Sabbatovskii K.G., Wang Y., Xu T., Nikonenko V.V. (2022). How Bulk and Surface Properties of Sulfonated Cation-Exchange Membranes Response to Their Exposure to Electric Current during Electrodialysis of a Ca^2+^ Containing Solution. J. Membr. Sci..

[94] de Freitas V., Carvalho E., Mateus N. (2003). Study of Carbohydrate Influence on Protein–Tannin Aggregation by Nephelometry. Food Chem..

[95] Alberti G., Narducci R., Sganappa M. (2008). Effects of Hydrothermal/Thermal Treatments on the Water-Uptake of Nafion Membranes and Relations with Changes of Conformation, Counter-Elastic Force and Tensile Modulus of the Matrix. J. Power Sources.

[96] Nebavskaya X., Sarapulova V., Butylskii D., Larchet C., Pismenskaya N. (2019). Electrochemical Properties of Homogeneous and Heterogeneous Anion Exchange Membranes Coated with Cation Exchange Polyelectrolyte. Membranes.

[97] Titorova V., Sabbatovskiy K., Sarapulova V., Kirichenko E., Sobolev V., Kirichenko K. (2020). Characterization of MK-40 Membrane Modified by Layers of Cation Exchange and Anion Exchange Polyelectrolytes. Membranes.

[98] Tsygurina K., Rybalkina O., Sabbatovskiy K., Kirichenko E., Sobolev V., Kirichenko K. (2021). Layer-by-Layer Coating of MK-40 Heterogeneous Membrane with Polyelectrolytes Creates Samples with Low Electrical Resistance and Weak Generation of H+ and OH−Ions. Membranes.

[99] Fidaleo M., Moresi M. (2006). Electrodialysis Applications in the Food Industry.

[100] Rybalkina O.A., Sharafan M.V., Nikonenko V.V., Pismenskaya N.D. (2022). Two Mechanisms of H+/OH− Ion Generation in Anion-Exchange Membrane Systems with Polybasic Acid Salt Solutions. J. Membr. Sci..

[101] Chandra A., Tadimeti J.G.D., Bhuvanesh E., Pathiwada D., Chattopadhyay S. (2018). Switching Selectivity of Carboxylic Acids and Associated Physico-Chemical Changes with PH during Electrodialysis of Ternary Mixtures. Sep. Purif. Technol..

[102] Helfferich F. (1962). Ion Exchange.

