# Supplementary material for: Comparison of the Electrodialysis Performance in Tartrate Stabilization of a Red Wine Using Aliphatic and Aromatic Commercial and Modified Ion-Exchange Membranes
Source: Membranes (Basel). 2023 Jan 9;13(1):84. doi: 10.3390/membranes13010084 (PMC9862077; doi:10.3390/membranes13010084)
Supplement: Supplementary file 1 [file membranes-13-00084-s001.zip › membranes-2144548-supplementary.pdf]

Supplementary materials

Comparison of the Electrodialysis Performance in Tartrate Stabilization of a Red Wine Using Aliphatic and Aromatic Commercial and Modified Ion-Exchange Membranes

Evgeniia Pasechnaya [1], Kseniia Tsygurina [1], Maria Ponomar [1], Daria Chuprynina [2], Victor Nikonenko [1] and Natalia Pismenskaya [1,*]

[1] Membrane Institute, Kuban State University, 350040 Krasnodar, Russia
[2] Department of Analytical Chemistry, Kuban State University, 350040 Krasnodar, Russia
* Correspondence: n_pismen@mail.ru

S1. Structure of the studied ion-exchange membranes

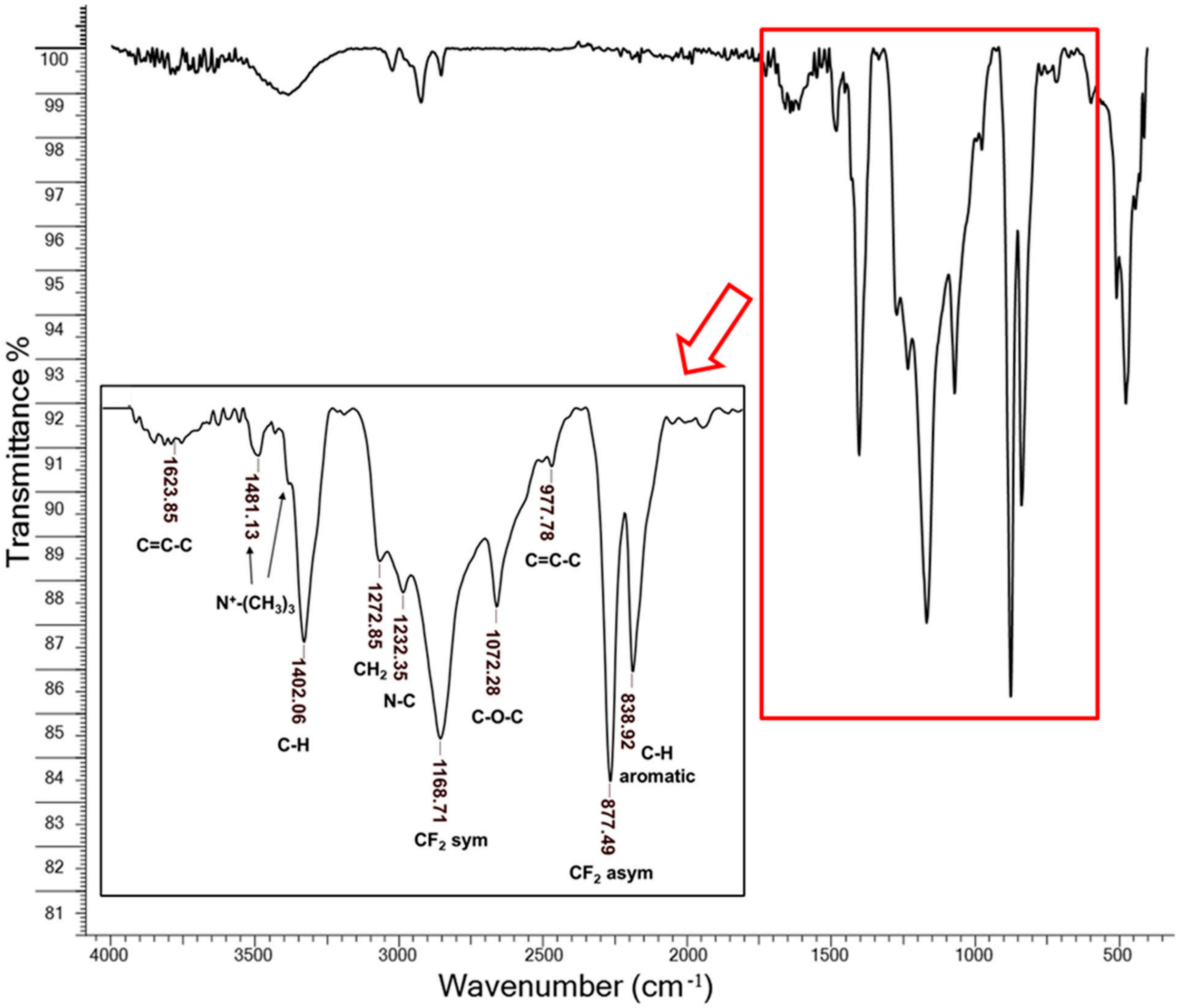

Figure S1. IR spectrum of the CJMA-3 membrane.

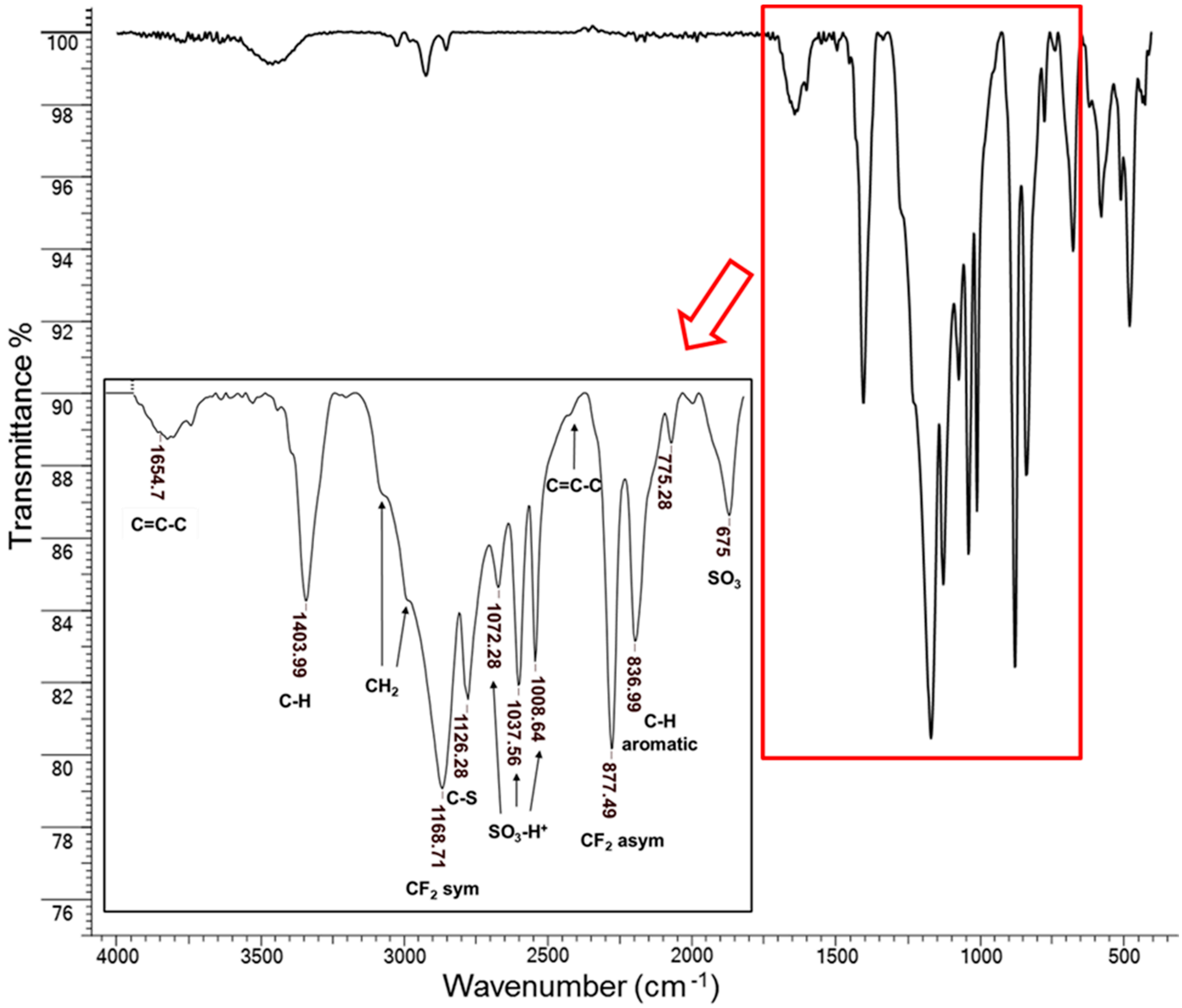

Figure S2. IR spectrum of the CJMC-3 membrane.

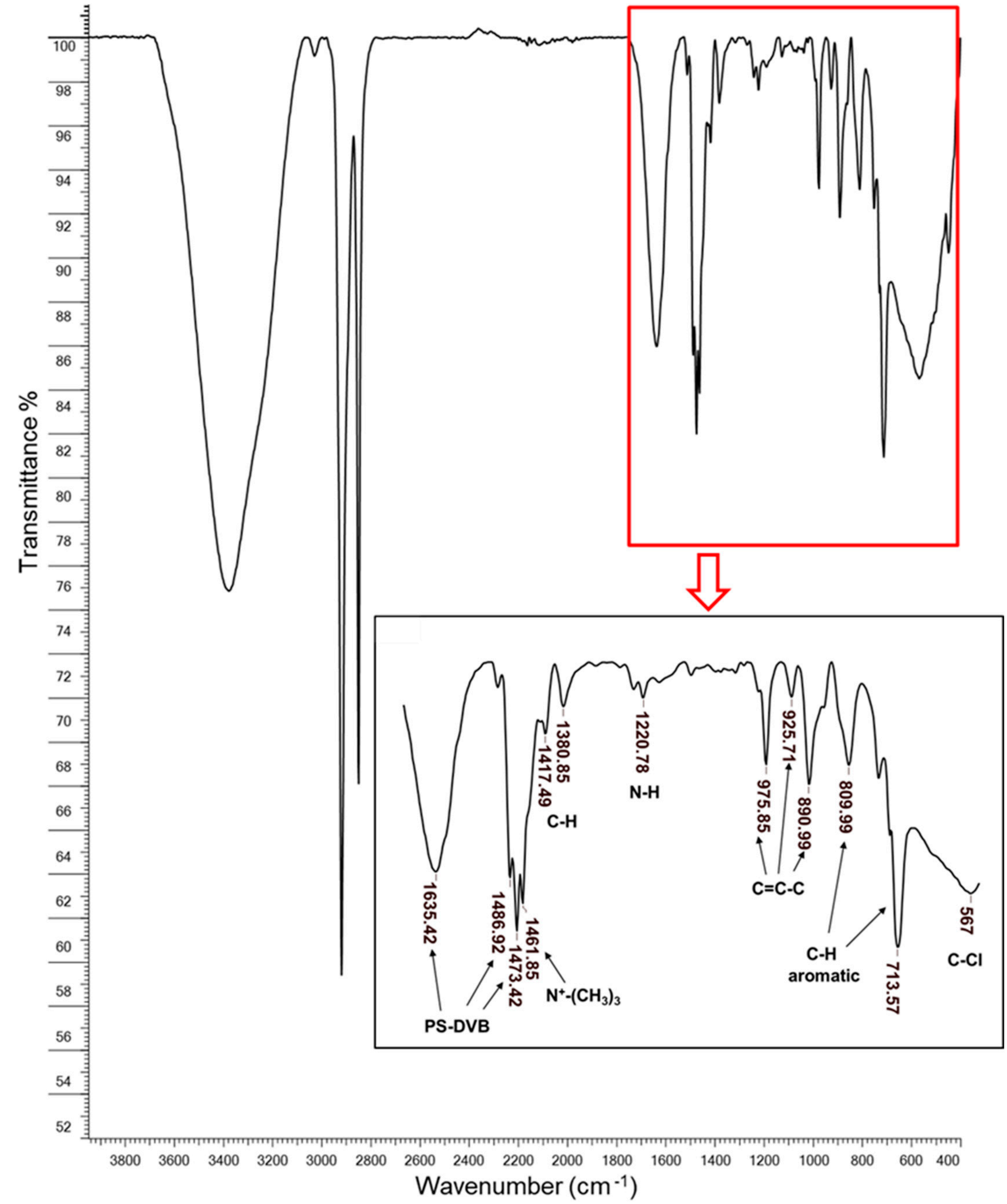

Figure S3. IR spectrum of the ASE membrane.

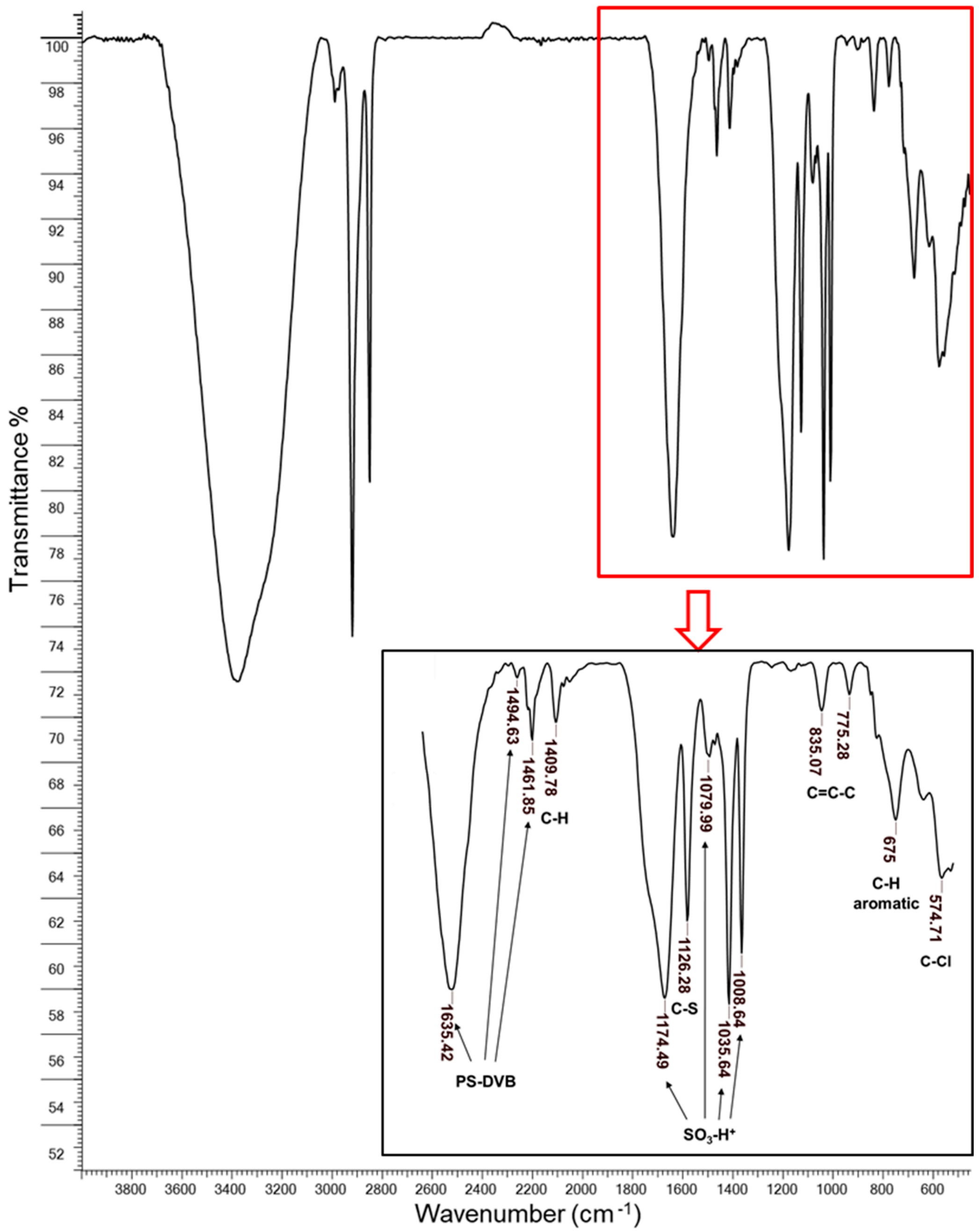

Figure S4. IR spectrum of the CSE membrane

S2. Structures of anthocyanins and their color

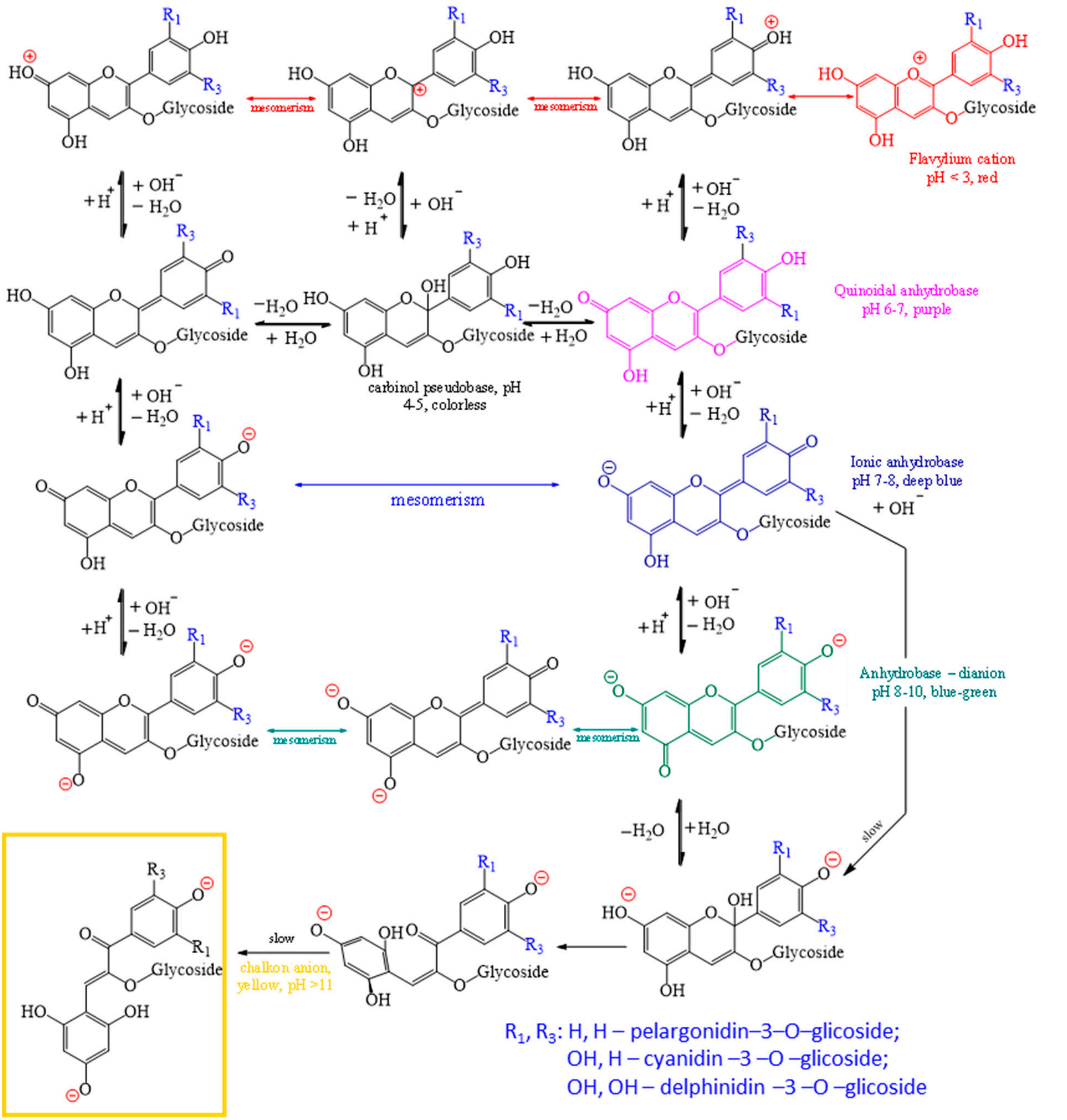

Figure S5. The structures of anthocyanins and their modifications depending on the pH of the medium. Adapted from [Ribéreau-Gayon et al., 2006]. *R*$_1$, *R*$_3$ are —H, or —OH, or —OCH$_3$ groups; *Glycoside* is glucose, rhamnose, arabinose or galactose.

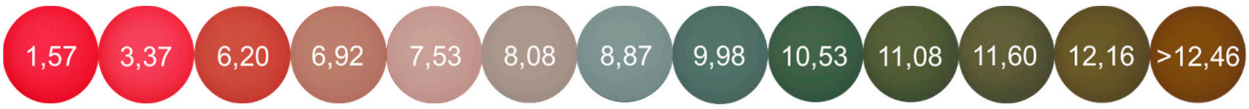

Figure S6. Effect of pH on the color of model solution. The pH values of the solutions are indicated for each color.
